# Fragmentation of extracellular ribosomes and tRNAs shapes the extracellular RNAome

**DOI:** 10.1093/nar/gkaa674

**Published:** 2020-08-12

**Authors:** Juan Pablo Tosar, Mercedes Segovia, Mauricio Castellano, Fabiana Gámbaro, Yasutoshi Akiyama, Pablo Fagúndez, Álvaro Olivera, Bruno Costa, Tania Possi, Marcelo Hill, Pavel Ivanov, Alfonso Cayota

**Affiliations:** Analytical Biochemistry Unit. Nuclear Research Center. Faculty of Science. Universidad de la República, Uruguay; Functional Genomics Unit, Institut Pasteur de Montevideo, Uruguay; Division of Rheumatology, Immunology and Allergy, Brigham and Women's Hospital, Boston, MA, USA; Department of Medicine, Harvard Medical School, Boston, MA, USA; Laboratory of Immunoregulation and Inflammation, Institut Pasteur de Montevideo, Uruguay. Immunobiology Department, Faculty of Medicine, Universidad de la República, Uruguay; Analytical Biochemistry Unit. Nuclear Research Center. Faculty of Science. Universidad de la República, Uruguay; Functional Genomics Unit, Institut Pasteur de Montevideo, Uruguay; Functional Genomics Unit, Institut Pasteur de Montevideo, Uruguay; Molecular Virology Laboratory, Nuclear Research Center. Faculty of Science. Universidad de la República, Uruguay; Division of Rheumatology, Immunology and Allergy, Brigham and Women's Hospital, Boston, MA, USA; Department of Medicine, Harvard Medical School, Boston, MA, USA; Analytical Biochemistry Unit. Nuclear Research Center. Faculty of Science. Universidad de la República, Uruguay; Functional Genomics Unit, Institut Pasteur de Montevideo, Uruguay; Centro Universitario Regional Este, Universidad de la República, Uruguay; Analytical Biochemistry Unit. Nuclear Research Center. Faculty of Science. Universidad de la República, Uruguay; Functional Genomics Unit, Institut Pasteur de Montevideo, Uruguay; Functional Genomics Unit, Institut Pasteur de Montevideo, Uruguay; Laboratory of Immunoregulation and Inflammation, Institut Pasteur de Montevideo, Uruguay. Immunobiology Department, Faculty of Medicine, Universidad de la República, Uruguay; Division of Rheumatology, Immunology and Allergy, Brigham and Women's Hospital, Boston, MA, USA; Department of Medicine, Harvard Medical School, Boston, MA, USA; The Broad Institute of Harvard and M.I.T., Cambridge, MA, USA; Functional Genomics Unit, Institut Pasteur de Montevideo, Uruguay; Department of Medicine, University Hospital, Universidad de la República, Uruguay

## Abstract

A major proportion of extracellular RNAs (exRNAs) do not copurify with extracellular vesicles (EVs) and remain in ultracentrifugation supernatants of cell-conditioned medium or mammalian blood serum. However, little is known about exRNAs beyond EVs. We have previously shown that the composition of the nonvesicular exRNA fraction is highly biased toward specific tRNA-derived fragments capable of forming RNase-protecting dimers. To solve the problem of stability in exRNA analysis, we developed a method based on sequencing the size exclusion chromatography (SEC) fractions of nonvesicular extracellular samples treated with RNase inhibitors (RI). This method revealed dramatic compositional changes in exRNA population when enzymatic RNA degradation was inhibited. We demonstrated the presence of ribosomes and full-length tRNAs in cell-conditioned medium of a variety of mammalian cell lines. Their fragmentation generates some small RNAs that are highly resistant to degradation. The extracellular biogenesis of some of the most abundant exRNAs demonstrates that extracellular abundance is not a reliable input to estimate RNA secretion rates. Finally, we showed that chromatographic fractions containing extracellular ribosomes are probably not silent from an immunological perspective and could possibly be decoded as damage-associated molecular patterns.

## INTRODUCTION

Extracellular RNA (exRNA) profiling in biofluids such as urine, plasma or serum is a promising approach for early disease detection and monitoring in minimally invasive liquid biopsies (1). Although plasma cell-free DNA analysis has proven powerful to detect cancer-associated mutations (2) or altered DNA methylation events (3), exRNA analysis has the potential to inform about transcript expression, post-transcriptional modifications and splicing variants (4). Additionally, it was reported that cells use exRNAs to communicate and reciprocally regulate their gene expression, even in distant tissues (5–7).

Because RNA is widely recognized as unstable in biofluids such as plasma due to their high RNase content (8), exRNAs are typically studied in the context of lipid membrane-containing extracellular vesicles (EVs) (7,9,10) or lipoprotein particles (LPPs) (11,12). Alternatively, exRNAs can achieve high extracellular stability by their association with proteins (13,14). However, extracellular soluble ribonucleoproteins remain the least studied exRNA carriers (15), with most attention thus far placed at the level of EVs.

Strikingly, a major proportion of extracellular small RNAs are found outside EVs (13,16). Furthermore, nonvesicular exRNA profiles are highly biased toward glycine and glutamic acid 5′ tRNA halves. This has been extensively documented both in cell culture media (16,17) and in biofluids such as urine, blood serum, saliva or cerebrospinal fluid (18–21). The abundance of these species in the extracellular, nonvesicular fraction (16,20,21)challenges the widespread belief that exRNAs are unstable when not present inside EVs and raise the question on the origin of their remarkable extracellular stability.

A possible answer to this question arises from our recent report that glycine and glutamic acid 5′ tRNA halves can form homo- or heterodimeric hybrids, which render them resistant to single-stranded RNases (22). The RNAs with predicted dimer-forming capacity are those of 30 or 31 nucleotides, which are slightly shorter than the 5′ tRNA halves generated by endonucleolytic cleavage of the anticodon loop (typically 34–35 nt) during the stress response (23–25). Interestingly, nonvesicular extracellular fractions are usually enriched in glycine and glutamic acid tRNA halves of precisely 30–31 nt (16,18), suggesting a causal link between extracellular stability and abundance (18). As a consequence, the enrichment of glycine and glutamic acid tRNA halves in extracellular samples does not necessarily imply that these sequences are released at higher rates from cells when compared to other small RNAs. Differential extracellular stability might as well produce the same outcome. Furthermore, the possibility that these fragments could be generated by extracellular fragmentation of tRNA precursors has not been sufficiently explored.

We hypothesized that extracellular RNA degradation is a major force shifting what cells release to the extracellular space toward those species with higher extracellular stability. Consequently, we speculated that conventional exRNA profiling fails to capture the complete set of RNAs released from cells to the extracellular space, frustrating attempts to infer RNA secretion mechanisms from comparisons between intracellular and extracellular RNA profiles. To study this, we compared exRNAs in cell-conditioned media obtained with or without addition of recombinant ribonuclease inhibitor (RI). Surprisingly, addition of RI greatly increased the complexity of exRNA profiles, stabilizing extracellular ribosomes and tRNAs which rapidly decay to rRNA- and tRNA-derived fragments in the absence of RNase inhibitors. Some of these fragments are highly stable and can accumulate in samples with high RNase activity, even when present outside EVs. Overall, we provide a dynamic and comprehensive characterization of the nonvesicular RNAome which can impact biomarker discovery in biofluids.

## MATERIALS AND METHODS

### Reagents

A full list of reagents including antibodies, primers and probes used in this study are reported in SI Materials and Methods.

### Preparation of cell-conditioned medium

Cells were cultured in DMEM + 10% fetal bovine serum (S+ medium) at 37°C and 5% CO_2_ or in different serum-free formulations as depicted in SI Materials and Methods. Recombinant ribonuclease inhibitor (RI; NEB) was added in selected experiments at a final concentration of 4–8 U/ml, either at the time of medium renewal or following collection of cell-conditioned media.

### Preparation of the nonvesicular extracellular fraction by ultracentrifugation

The cell-condtioned medium already spinned at 2000 × g was centrifuged for 2.5 h at 100 000 × g and 4°C in a Beckman Coulter Optima XPN-90 ultracentrifuge using a SW40 Ti rotor. The supernatant was concentrated to ∼250 μl with 10 000 MWCO ultrafiltration units (Vivaspin 20, Sartorious Stedim Biotech) and subjected to size-exclusion chromatography or RNA extraction with TRIzol (according to manufacturer's instructions).

### Size exclusion chromatography

The 100 000 × g supernatants of the cell-conditioned medium were concentrated by ultrafiltration by successive dilutions with PBS to remove phenol red and small molecules. Concentrated EV-depleted fractions (500 μl) were injected in a Superdex S200 10/300 column (Amersham, GE) and size exlusion chromatography (SEC) was performed at 0.9 ml/min in 0.22 μm-filtered 1× PBS with an Äkta Pure (GE healthcare) FPLC system. All samples were centrifuged at 16 000 × g for 10 min at 4°C before injection in the columns. Fractions of 0.25 ml were collected while monitoring the absorbance at 260 and 280 nm. Chromatograms were deconvoluted *in silico* using empirically determined 260/280 ratios from pure bovine serum albumin (BSA) and synthetic tRNA^Gly^_GCC_ 5′ halves (of 30 nt).

### Sequencing of small RNAs

RNA was purified from selected chromatographic peaks using TRIzol LS and following manufacturer's instructions. The obtained RNA was diluted in 8 μl of ultra-pure RNase-free water, and 7 μl were used as input for NGS library preparation using the NEBNext Small RNA Library Prep Set for Illumina (New England Biolabs). Sequencing was performed in a MiSeq benchtop sequencer for 200 cycles. The analysis was performed as depicted in SI Materials and Methods. FastQ files containing sequences of at least 15 nt after 3’ adapter identification and removal are available at NCBI SRA.

### Northern blotting

Northern blots (10% denaturing gels transferred to positively-charged nylon membranes) were performed based on digoxigenin-labeled DNA probes and an alkaline phosphatase-labeled anti-digoxigenin antibody (Roche). Signals were visualized with CDP-Star ready-to-use (Roche) and detected using ChemiDoc imaging system (BioRad). A detailed protocol and probe sequences are provided in SI Materials and Methods.

### Differentiation of dendritic cells and flow-cytometry.

Adherent mouse bone marrow-derived dendritic cells (BMDC) were prepared as described in (26). Selected chromatographic fractions or synthetic RNAs were added to 1 × 10^6^ BMDCs. At *t* = 24 hs, cells were harvested and analyzed by flow cytometry using a CyAn ADP Analyzer (Dako). A detailed protocol is provided in SI Materials and Methods.

## RESULTS

### Inhibition of extracellular ribonucleases dramatically alters exRNA profiles

We have previously shown that the most abundant non-vesicular exRNAs have the capacity to form homo- and heterodimers and are therefore highly stable against the action of extracellular RNases (16,22). Consequently, we reasoned that conventional experimental approaches to assess exRNAs could be biased toward the most stable entities and miss most of the RNAs actually released from cells due to their low extracellular half-life.

In order to understand exRNA dynamics and capture both stable and unstable RNAs, we developed a method based on size-exclusion chromatography (SEC) fractionation of RNase inhibitor (RI)-treated cell-conditioned medium (RI-SEC-seq). Briefly, MCF-7 cell-conditioned medium (CCM) was treated (or not) with a recombinant RNase A family inhibitor, and EVs were later removed by ultracentrifugation to obtain the ‘EV-depleted’ extracellular fraction. Next, SEC separation was used to identify major RNA populations, which were subjected to standard small RNA sequencing procedures.

**Figure 1.**
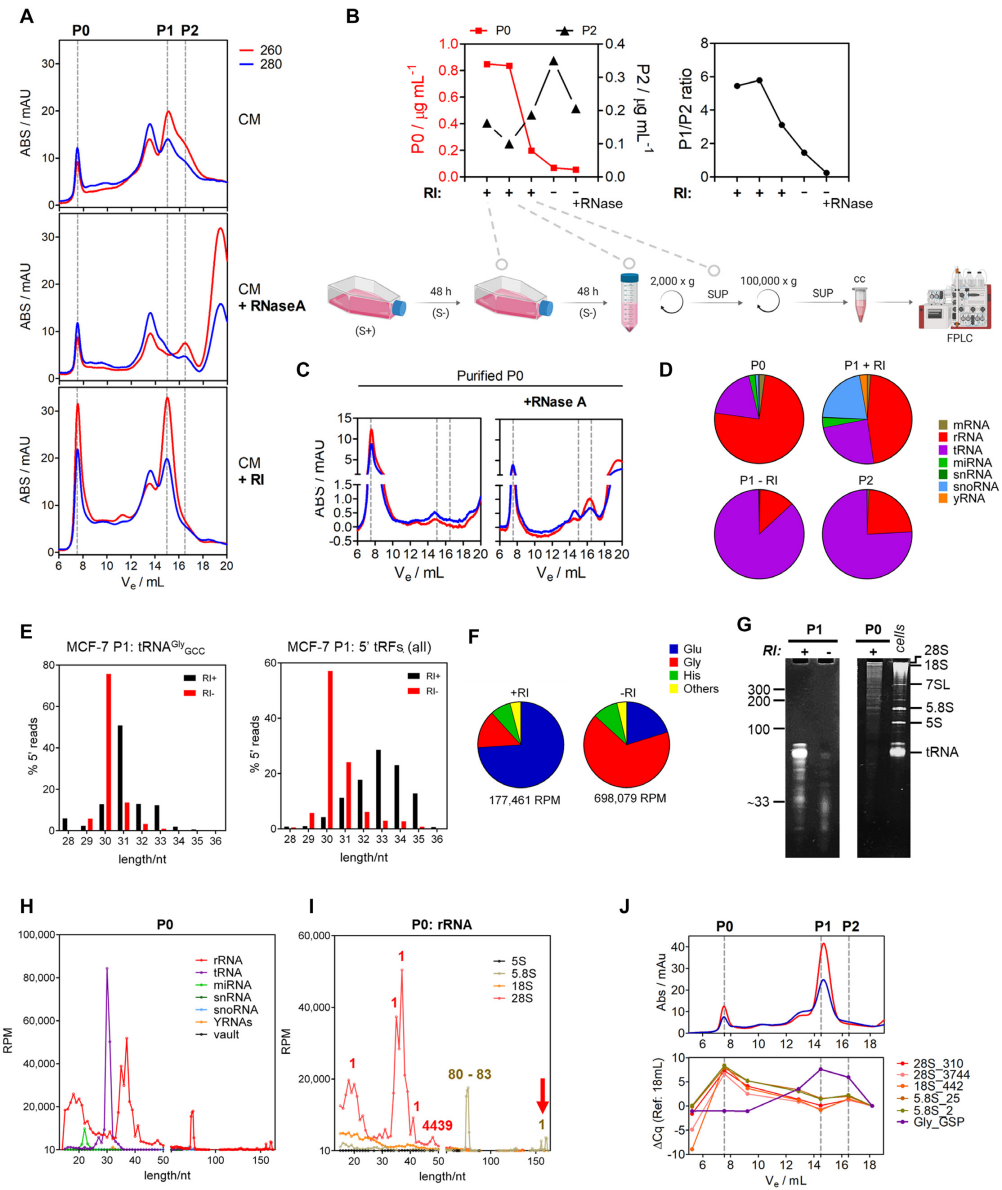
RNase inhibition stabilizes full-length extracellular rRNAs and tRNAs. (**A**) Size exclusion chromatography (SEC) of 100 000 × g supernatants of MCF-7 cell-conditioned medium (CM) following addition of RNase A (middle) or Ribonuclease Inhibitor (RI, bottom). Red, Blue: absorbance at 260 and 280 nm, respectively. (**B**) The earlier RI was added to the CCM during sample preparation, the higher the P0/P2 (left) and P1/P2 (right) ratios. S+, S–: serum-containing and serum-free media, respectively. SUP: supernatant. (**C**) The P0 peak was purified after SEC and treated with RNase A, which partially reconstituted the P2 peak. (**D**) Pie charts showing relative representations of different RNA biotypes in RI-SEC-seq datasets. (**E**) Size distribution of reads mapping to the 5’ half of glycine tRNA (left) or to all tRNAs (right) in the P1 peak from MCF-7 cells either with (black) or without (red) addition of RI. (**F**) Relative representation of reads mapping to different tRNA isoacceptors in the P1 peak of cells treated (top) or not (bottom) with RI. (**G**) Analysis of the P1 peak either with (+) or without (−) RI treatment (left gel) or the P0 peak (right gel) in a denaturing polyacrylamide gel. (**H**) Size distribution of small RNA sequencing reads mapping to different ncRNAs (see legend) in the P0 peak of MCF-7 cells. RPM: reads per million mapped reads. (**I**) As in (H) but showing only the reads aligning to rRNAs. Numbers indicate starting position of most reads. (**J**) Amplification of different rRNA regions by random-primed RT-qPCR in fractions collected after SEC. Gly_GSP: amplification of glycine 5’ halves using a gene-specific primer during RT. Numbers after underscores represent the 5’ position of the expected amplicon.

Under basal (-RI) conditions, chromatographic analysis of the EV-depleted fraction of MCF-7 CCM consistently showed two peaks with Abs 260 > Abs 280 which we termed P1 (*V*_e_ = 15.0 ml) and P2 (*V*_e_ = 16.5 ml) (Figure 1 A; top). The elution volume of P1 corresponds to that obtained when injecting yeast full-length tRNA^Phe^ or RNA dimers of 30 nt (Supplementary Figure S1, A and (22)). In contrast, synthetic ssRNAs of 30 nt elute in P2. Treatment of the CCM with exogenous RNase A prior to SEC depleted the P1 peak and degradation products were evident with *V*_e_ ≥ 19 ml (Figure 1A, middle). However, absorbance in the P2 region was only modestly affected. Addition of the recombinant RNase A-family inhibitor (RI) to the CCM precluded the formation of P2 (Figure 1A, bottom), demonstrating that the P2 peak also contains RNA. In this situation, the P1 peak was still prominent, but similar amounts of nucleic acids now eluted in the exclusion volume of the column (*V*_e_ = 7.5 ml; apparent MW ≥ 800 kDa). For simplicity, we will term this new peak ‘P0’.

The high MW RNA-containing complex in P0 was mutually exclusive with the P2 peak (Figure 1B). Furthermore, P2 could be reconstituted by RNase A treatment of the purified P0 fraction (Figure 1C). Taken together, these results suggest that P2 contains a highly resistant small RNA population formed by partial fragmentation of long RNAs within P0 (and possibly P1 as well).

### RI-SEC-seq offers an unbiased and dynamic view of the extracellular RNAome.

Small RNA sequencing of the different chromatographic fractions showed that P0 is mainly composed of rRNAs (Figure 1D). Sequencing of P1 obtained with or without addition of RI was previously published by our group (22) and showed a predominance of 5′ tRNA halves derived from tRNA^Gly^_GCC_ and tRNA^Glu^_CUC_. However, tRNA-derived fragments in the ‘+RI’ library tended to be longer than in ‘–RI’ (33–35 versus 30–31) (Figure 1E and Supplementary Figure S1B). On top of that, PI – RI was highly enriched in glycine tRNA halves of 30 nt when compared to PI + RI (Figure 1F). The ability of these fragments to form RNase-resistant homodimers is consistent with their detection in the absence of RI and their elution in the P1 peak. Interestingly, addition of RI recovered a higher diversity of small RNAs in the P1 peak, including fragments of rRNA and full-length snoRNAs (Supplementary Figure S1C). It is remarkable that the main rRNA-derived fragments and snoRNAs sequenced in P1 + RI are known to interact (Supplementary Figure S1D). Thus, it is possible to speculate that dsRNA hybrids tend to accumulate in extracellular nonvesicular samples due to their intrinsic resistance to extracellular single-stranded ribonucleases. Nevertheless, the stabilities of glycine tRNA halves are outstanding since these are virtually the only species identified in the absence of RI.

Sequencing of full-length mature tRNAs is challenging due to the abundance of modified ribonucleotides and their strong self-complementarity. Therefore, specific protocols for tRNA sequencing and analysis have been developed in recent years (27–30). Despite using conventional ligation-based library preparation methods, we obtained a low but nonnegligible number of reads corresponding to two full-length tRNA^Glu^ isoacceptors (Supplementary Figure S1E). These sequences were exclusively detected in the ‘P1 + RI’ library and contained the 3′ terminal nontemplated CCA addition indicative of mature tRNAs. Analysis of the P1 + RI fraction in a denaturing RNA gel showed that, indeed, most RNAs migrated like full length tRNAs (Figure 1, G). In contrast, the majority of RNAs in ‘P1 –RI’ were < 33 nt, consistent with tRNA halves. Altogether, these results suggest that exRNA profiles are biased toward highly stable tRNA halves by the action of extracellular ribonucleases and they do not necessarily reflect the composition of RNAs actually released by cells.

The P0 peak contains mostly high-MW RNA, as evidenced by denaturing gel electrophoresis (Figure 1, G) and because reinjection of the purified RNA from the P0 peak still eluted in the void volume (Figure 1C). Small RNA sequencing showed the same tRNA-derived fragments found in P1, but overwhelmingly higher levels of rRNA fragments (Figure 1D, H–I). The majority of these were 28S rRNA 5′ fragments of 20–40 nucleotides. Our size-selected libraries were too short (<200 nt) to capture the complete 28S (5070 nt) or 18S (1869 nt) rRNAs, although sequences spanning the entire 28S rRNA were detectable (Supplementary Figure S1F). Nevertheless, the entire 5.8S rRNA could be read (156 nt; Figure 1I and Supplementary Figure S1G).

To demonstrate the presence of full-length rRNAs in P0 we performed random-primed reverse transcription and qPCR amplification with primers spanning different regions of 28S, 18S and 5.8S rRNAs (Figure 1J). All primer sets amplified comparably and exclusively in P0, while tRNA^Gly^_GCC_ 5′ tRNA halves peaked in P1 and P2 as expected. This evidence, together with sequencing of the entire 5.8S rRNA, strongly suggests the release of intact rRNAs from both ribosomal subunits to the extracellular space and outside EVs.

### Release of rRNAs and tRNAs occurs promptly after 30 seconds of medium renewal

**Figure 2.**
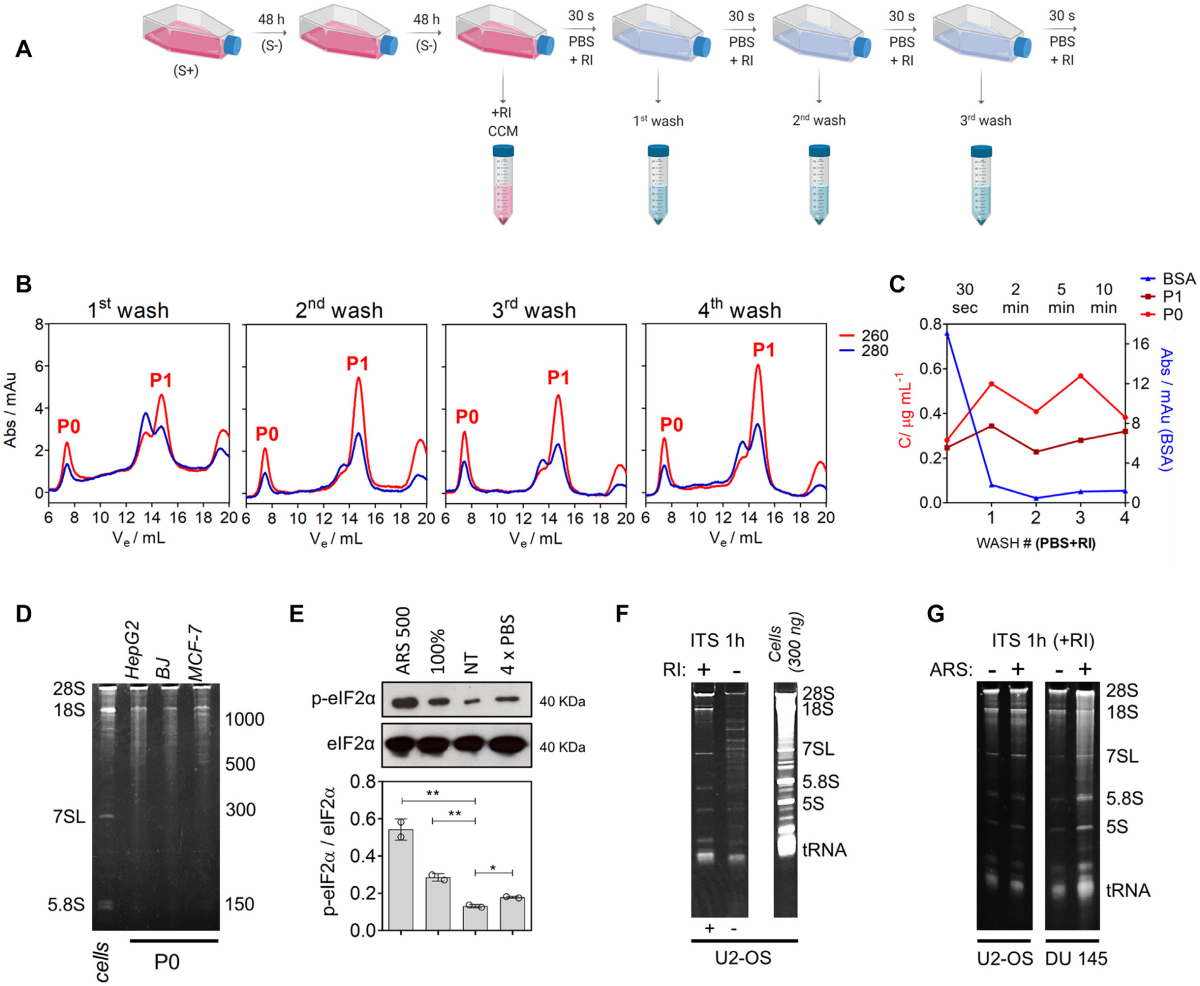
rRNAs and tRNAs are rapidly released to the extracellular space. (**A**) Schematic representation of the experimental protocol used in panels B–E. S+: DMEM + 10% FBS. S–: serum-free MEGM medium. (**B**) RNA analysis by SEC in PBS washes #1 to #4 of MCF-7 cells. Conditions identical to those used in Figure 1. (**C**) Variation of RNA concentration corresponding to the P0 (light red) and P1 (dark red) peaks in PBS washes #1 to #4. The variation in the Abs 280 nm of the BSA peak is plotted in the right axis. Initial values correspond to those present in the CCM. (**D**) Denaturing electrophoresis (6% PAGE) of the concentrated P0 peak from the fourth PBS wash of HepG2, BJ and MCF-7 cell lines. No RNA purification was performed. ‘Cells’: MCF-7 RNA lysate. (**E**) Analysis of eIF2 alpha phosphorylation (Wblot) in nontreated (NT) MCF-7 cells, in cells exposed to four consecutive PBS washes (4× PBS), in cells cultured after confluency (100%) or exposed to 500 μM sodium arsenite for 1 h (ARS 500). Bottom: densitometry analysis in two independent biological replicates of the experiment. (**F**) Denaturing electrophoresis (10% PAGE) of purified extracellular RNA from U2-OS cells incubated for 1 h in a serum-free medium (DMEM + ITS, ± RI; 5 × 10^6^ cells), ran alongside 300 ng of purified U2-OS intracellular RNA. (**G**) Purified RNA from U2-OS (left) or DU 145 (right) cell-conditioned medium (ITS + RI, 1 h) obtained in the presence (+) or absence (−) of 200 μM sodium arsenite.

To clearly differentiate between RNAs directly released from cells and fragments generated by extracellular processing of longer precursors, we combined RI treatment with exRNA profiling shortly after medium renewal. To do this, we performed four sequential washes of cells with PBS and analyzed the RNA content in the cell-conditioned PBS, with a conditioning time as short as 30 s (Figure 2A). Strikingly, both the rRNA-associated P0 and the tRNA-associated P1 peaks were consistently detected in the four washes (Figure 2B). Similar results were obtained when washing with serum-free chemically defined growth medium (MEGM; Supplementary Figure S2A), albeit with a higher wash-to-wash variability. Thus, detection of rRNAs and tRNAs in the fourth PBS or MEGM wash was not a consequence of carry-over from previously longer incubations. Instead, fast release of these RNAs occurred every time the cells were washed. Increasing incubation time from 30 s to 10 min did not increase extracellular RNA levels (Figure 2C). This phenomenon was highly reproducible in a variety of adherent cell lines, including primary normal cell lines such as BJ fibroblasts (Figure 2D and Supplementary Figure S2B).

As expected, the vast majority of cells were not affected by the washing protocol and retained their anchorage (Supplementary Figure S2C). Induction of cell stress was not evident based on eIF2α phosphorylation levels (Figure 2E). However, floating nuclei were observable after the washes when serum-free media or different buffers were used (data not shown), questioning the source of the exRNAs detected in the media. Estimated RNA levels in each wash were 0.1–0.5% of the total RNA present in the culture.

### Extracellular nonvesicular RNAs mirror the intracellular transcriptome

To distinguish between selective and nonselective release of cytoplasmic contents, we scaled up our cultures in an attempt to detect less abundant nonvesicular exRNAs. Surprisingly, denaturing PAGE analysis of TRIzol-purified RNA from extracellular, RI-treated fractions showed all RNA bands typically found in cellular extracts and respecting their relative intensities (Figure 2F). This was observed also in the DU145 cell line which is deficient in ATG5 and therefore in autophagy (31) (Figure 2G) suggesting that the release mechanism operating herein is unrelated to the recently described autophagy-dependent secretion of cytoplasmic nucleic acids (32).

### Identification of ribosomes and oligoribosomes in the extracellular space

**Figure 3.**
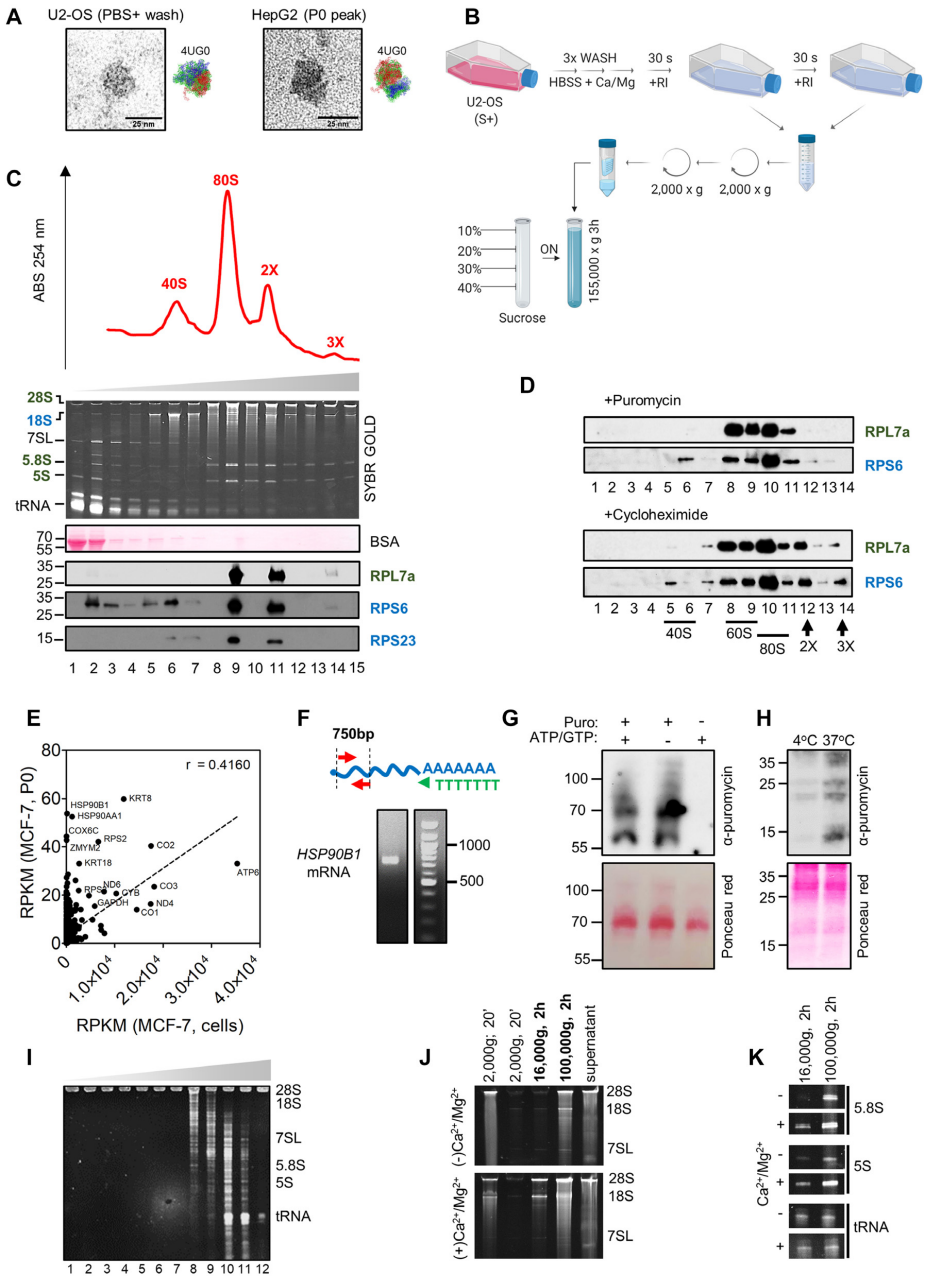
The extracellular space contains ribosomes. (**A**) Negative-stain TEM images of ribosome-like particles in concentrated cell-conditioned PBS or chromatographic P0 peak from U2-OS and Hep G2 cells, respectively. Scale bar: 25 nm. A Cryo-EM structure of the human ribosome (PDB: 4UG0) is shown for comparison, with 28S and 18S rRNAs colored red and blue, respectively. (**B**) Schematic representation of the experimental protocol used in panels C and D. (**C**) Velocity gradient (10–40% sucrose, 3 h at 155 000 × g) of cell-conditioned HBSS (plus divalent cations and RI) from U2-OS cells. Fractions of 0.5 ml were taken out from the top of the tube and treated with TRIzol. RNA was analyzed in a denaturing 10% PAGE and proteins were analyzed by western blot using antibodies specific to human ribosomal proteins. (**D**) Same as (C), but cells were treated with puromycin (2 μg /ml; 10 min) or cycloheximide (10 μg/ml; 30 min) before washes. (**E**) Analysis of mRNAs in the P0 peak of MCF-7 cells. Relative abundances (expressed as reads per kilobase of transcript, per million mapped reads, RPKM) assume random fragmentation of parental mRNAs. Intracellular abundances in MCF-7 cells were based on transcriptomic data from the Human Protein Atlas. (**F**) RT-PCR of human HSP90B1 mRNA with an oligo-dT primer and PCR primers complementary to exons 5 and 9 (2229 bp from 3’ end). (**G**) U2-OS cell-conditioned buffer (+RI, + divalent cations) was concentrated by ultrafiltration and treated with 5 μg/ml puromycin for 2 h at 37°C with or without addition or ATP/GTP. Extracted proteins were analyzed by Western blot with an anti-puromycin antibody. (**H**) Same as (G), but incubating samples at either 37°C or 4°C. (**I**) Isopycnic centrifugation in a 12–36% iodixanol gradient of the concentrated fourth wash of U2-OS cells (HBSS+). Twelve fractions of 1 ml were collected from the top of the tube. Densities in each fraction were comparable to ref 32. (**J, K**) U2-OS cells were washed with HBSS with/without divalent ions for 30 s. Samples were centrifuged twice at 2000 × g, followed by sequential ultracentrifugation. Pellets were resuspended in water and, together with the concentrated 100 000 × g supernatant, ethanol precipitated, resuspended, and analyzed by denaturing electrophoresis. Panels I and J show different regions of the same gel.

We could identify features with size and morphology consistent with the presence of ribosomal particles by negative-stain transmission electron microscopy of the concentrated P0 peak or unfractionated PBS washes of different adherent cell lines (Figure 3A). To confirm the presence of extracellular ribosomes, we resort to study concentrated extracellular fractions by velocity sedimentation in sucrose gradients optimized for polysome preparations (Figure 3B). Analysis of the velocity gradients showed three clearly defined 254 nm peaks in the region corresponding to ribosomal subunits or ribosomes (Figure 3C). These peaks corresponded to the small ribosomal subunit 40S (low levels of 28S, 5.8S and 5S rRNA, high levels of 18S rRNA, low levels of RPL7a, high levels of RPS6), 80S monosomes (all rRNAs and all ribosomal proteins detectable with the expected stoichiometry), and, considering a lack of detectable ribosomal proteins in fraction #10 and their reappearance in fraction #11, two ribosomes (2×). Interestingly, a small 254 nm peak in fraction #14 was accompanied by a faint but detectable band for RPL7a and RPS6 and was indicative of oligoribosomes or polysomes. These were further stabilized by treating cells with the translation elongation blocker cycloheximide (but not with the premature terminator puromycin) straight before HBSS+ washes (Figure 3D).

Because polysomes are formed by ribosomes sitting on messenger RNAs, we wondered whether the extracellular fractions also contained mRNAs. A reanalysis of our small RNA sequencing in the P0 peak from MCF-7 cells revealed a variety of reads unambiguously mapping to coding sequences. Considering only reads of 19 nt or longer, we could detect 125 unique mRNAs supported by at least five counts each. In many cases, reads mapped to different exons and were long enough (30–40 nt) to discard the possibility of ambiguous mapping. Once again, their extracellular representation was positively correlated (*r* = 0.416, *P* < 0.0001) to their expected intracellular levels, and included mRNAs transcribed from the mitochondrial genome (Figure 3E). One of the most abundantly detected mRNAs in our sequencing study, HSP90B1, could be amplified from the P0 peak of different cells using an oligo dT reverse transcription primer and PCR primers aligning to its 5′ end (Figure 3F). Thus, complete, nondegraded mRNAs are detectable in the extracellular space and co-purify with extracellular ribosomes. Long reads representing full-length, spliced mRNAs were also identified by direct RNA sequencing of extracellular samples using an Oxford Nanopore MinION sequencer (data not shown). RNA inputs were too low to obtain a reliable and representative description of extracellular mRNAs. Nevertheless, the presence of full-length mRNAs in these extracellular fractions is strongly supported.

Next, we addressed whether extracellular ribosomes/polysomes contained attached polypeptides. To do so, we incubated concentrated extracellular samples with puromycin (an analog of the 3′ end of aminoacyl-tRNA) and evaluated incorporation of the antibiotic to nascent peptides by Western blot. Interestingly, puromycin incorporation was independent of added ATP/GTP (Figure 3G) but was dependent on temperature (Figure 3H), consistent with the energy independency (33) but temperature dependency (34) of the puromycylation reaction. These results also suggest that extracellular ribosomes contain pre-formed polypeptides (considering elongation unlikely under these experimental conditions) and were therefore engaged in translation before their release to the extracellular space. To the best of our knowledge, this is the first evidence of the peptidyl transferase reaction occurring in extracellular samples obtained without deliberated cell lysis steps.

### Nonvesicular ribosomal RNAs co-purify with extracellular vesicles

Isopycnic centrifugation of exRNAs obtained after short washes of U2-OS cells with HBSS+ confirmed that virtually all RNAs detectable by SYBR gold staining were not associated with EVs (Figure 3I). However, sequential ultracentrifugation of cell-conditioned HBSS (either with or without divalent cations) showed sedimentation of rRNA- and tRNA-containing complexes at speeds usually used to purify small EVs (100 000 × g; Figure 3J). Strikingly, presence of divalent ions favored rRNA sedimentation at speeds typically used to collect large EVs (16 000 × g). This effect was not observed for tRNAs (Figure 3K). These observations are consistent with the involvement of magnesium ions in the stabilization of ribosomes (35) and demonstrate that the most frequently used method for EV purification (i.e. ultracentrifugation) does not allow separation of EVs from nondegraded extracellular ribosomes.

### Extracellular biogenesis of extracellular tRNA halves

As shown above (Figure 1), full-length tRNAs comprise the majority of RNAs present in the P1 peak in RI-treated CCM, but this profile shifted toward RNase-resistant glycine tRNA halves (30–31 nt) in the absence of RI. One possibility is that these tRNA halves were released directly from cells and accumulated because of their resistance to degradation. Alternatively, they could be generated by the fragmentation of extracellular tRNAs.

To distinguish between both possibilities, we developed different protocols where the activity of extracellular ribonucleases could be experimentally modulated. These protocols allowed us to obtain exRNAs with virtually no degradation (short washes in the presence of RI), or in the presence of FBS, which contains highly active RNase A.

**Figure 4.**
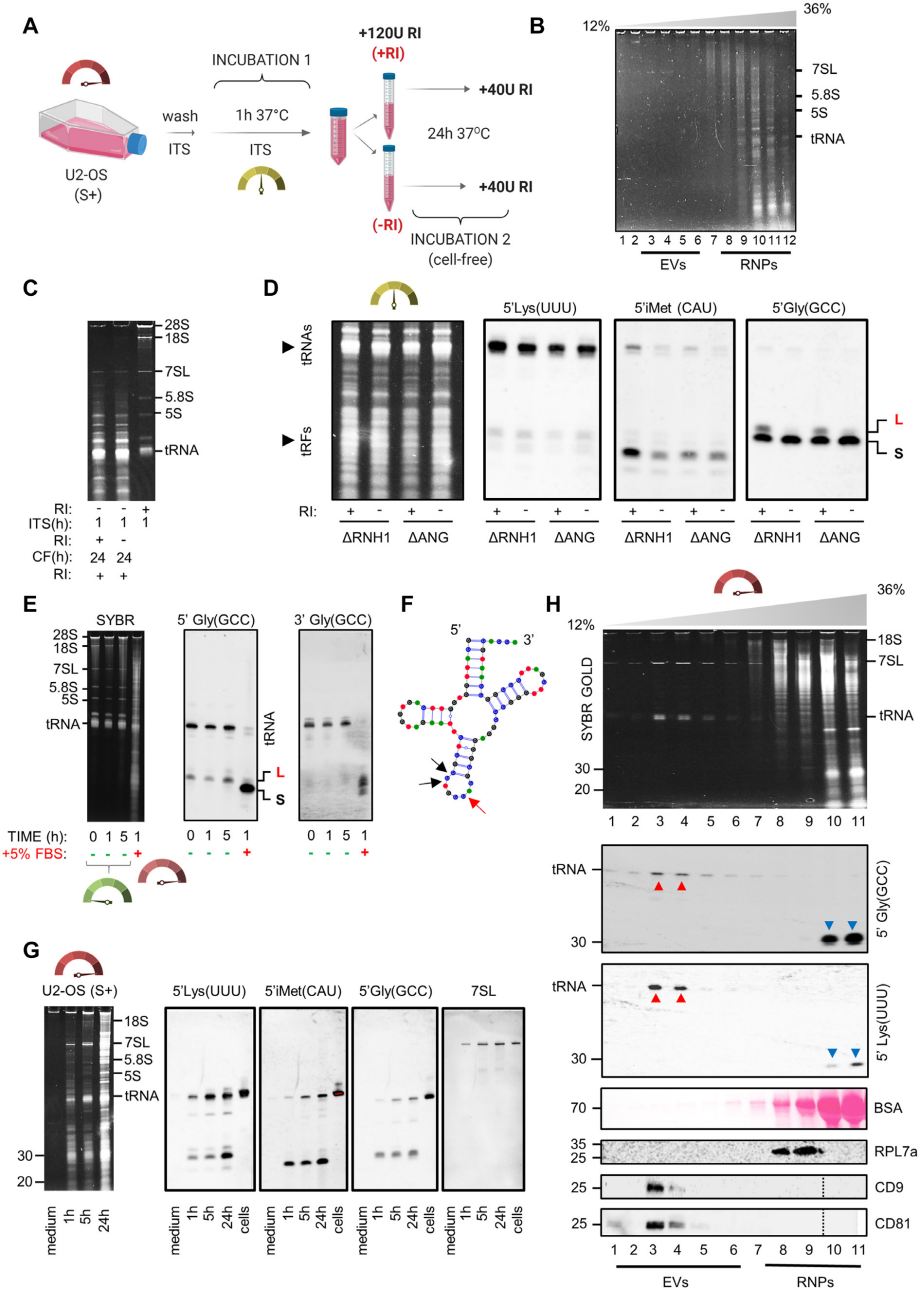
Extracellular tRNAs are processed to extracellular tRNA-derived fragments. (**A**) Schematic representation of the experimental setup used in panels B–D. (**B**) Iodixanol gradient showing most exRNAs were present in the nonvesicular fractions (RNPs) in the input sample (before cell-free processing). (**C**) Analysis of exRNAs by denaturing electrophoresis before and after the cell-free (CF) maturation step. (**D**) Northern blot analysis with probes targeting the 5’ region of different tRNAs in samples obtained as explained in (A). L: 5’ tRNA halves of 33–34 nt; S: 5’ tRNA halves of 30–31 nt. (**E**) Samples were obtained after a short (30 s) wash of U2-OS cells with HBSS+ (without RI addition), and incubated cell-free at 37°C for 0, 1 or 5 h, or for 1 h after addition of S+ medium in a 1:1 ratio. Northern blot was performed with two different probes targeting both halves of tRNA^Gly^_GCC_. (**F**) Cloverleaf representation of glycine tRNA (GCC anticodon, isodecoder #2) with red arrow showing predicted cleavage at the anticodon loop (sequence CpCpA), rendering a 33 or 34 nt 5’ fragment, and black arrows showing a putative alternative cleavage site (sequence CpCpU), generating 30–31 nt 5’ fragments. (**G**) Analysis of exRNAs in U2-OS CCM (1, 5 and 24 h, S+ medium). The concentrated nonconditioned medium was run as a control. Northern blot was done with the same probes as in panel (D), plus a 7SL RNA-specific probe. (**H**) Isopycnic centrifugation of U2-OS CCM (*t* = 24 h in S+ medium). Color code (dials): green (short wash with RI; low RNase activity), yellow (endogenous RNases, released during 1 h incubation in serum-free medium without RI), red (FBS-derived RNases).

As an intermediate situation between both extremes, a third protocol was optimized where cells were washed and incubated in serum-free medium (DMEM + ITS supplement, hereafter ‘ITS’) for 1 h and in the absence of RI (protocol 3, SI Materials and Methods). Under these experimental conditions, RNase activity relies mostly on endogenous secreted ribonucleases. Cell-conditioned medium was later incubated at 37°C for 24 h in order to amplify the effects of endogenous RNases (‘cell-free maturation step’, Figure 4A).

Iodixanol density gradient centrifugation confirmed that most of the RNAs were present in the nonvesicular fractions after an incubation of U2-OS cells in ITS for 1 h (Figure 4B). Endogenous RNase activity was evident when comparing exRNA profiles with or without RI, irrespectively of the cell-free maturation step (Figures 2F and 4C). When including this step, rRNA bands were no longer detectable, but the tRNAs were affected to a much lower extent (Figure 4C). Surprisingly, Northern blot analysis showed marked differences in tRNA stability (with tRNA^Lys^_UUU_ > tRNA^iMet^_CAU_ > tRNA^Gly^_GCC_), which were inversely correlated to their corresponding 5′ tRNA halves (Figure 4D). This strongly suggest that extracellular tRNA halves are generated by extracellular cleavage of tRNAs. This cleavage is dependent on extracellular RNase A family members because it can be modulated by exogenously added RI, but Angiogenin is dispensable or redundant based on almost identical results in wild-type (Figure 4C) or ANG knock-out cells (Figure 4D).

Two bands were observed in the case of glycine 5′ tRNA halves. These were 33–35 nt (i.e. 5′ tRNA halves cleaved at the anticodon loop) or 30–31 nt (Figure 4D and Supplementary Figure S3A). For simplicity, we will call these fragments long tRNA halves (L-tRNAh) and short tRNA halves (S-tRNAh), respectively. Both species are detectable inside U2-OS cells stressed with sodium arsenite but L-tRNAh are produced at much higher levels (36). In contrast, only the S-tRNAh are predicted to form RNase-resistant homodimers according to our previous study (22). Strikingly, only the S-tRNAh was detectable in the extracellular milieu in the absence of RI (Figure 4D) confirming the enhanced extracellular stability of this specific fragment.

Five prime tRNA halves were termed ‘stress-induced tRNA-derived fragments’ or tiRNAs (24) because their production is induced by cellular stress. Loading of one microgram of intracellular RNA from U2-OS cells did not show production of these tRNA halves or tiRNAs above the assay's sensitivity (Supplementary Figure S3, A). Despite the full-length tRNA^Gly^_GCC_ was not detectable after 24 h of cell-free maturation of the CCM (Figure 4D), the full-length tRNA as well as the L-tRNAh and S-tRNAh were clearly present if this cell-free maturation step was omitted (Supplementary Figure S3A). Interestingly, 3′ tRNA-derived fragments were also detectable in the same samples although they are rarely found intracellularly, even in arsenite-treated U2-OS cells (24). The higher the degradation state of the exRNA population, the higher the fragment-to-tRNA ratio for both 5′ and 3′ fragments (Supplementary Figure S3B).

To obtain direct evidence for the conversion of extracellular tRNAs into tRNA halves, we briefly washed U2-OS cells with HBSS+ in the absence of RI and divided the cell-conditioned buffer into four aliquots. These aliquots were incubated for 0, 1 or 5 h at 37°C before addition of RI and subsequent analysis by Northern blot (Figure 4E). The fourth aliquot was mixed 1:1 with S+ medium (to obtain a final serum concentration of 5%) and incubated for 1 h at 37°C. The full-length tRNA^Gly^_GCC_ and the L-tRNAh (but not the S-tRNAh) were present at *t* = 0. Incubation for 5 h at 37°C showed a slight decrease in the intensity of the full-length band and a concomitant increase in L-tRNAh. Strikingly, incubation for only 1 h in the presence of serum (RNase-rich sample) entirely converted the full-length tRNA band to S-tRNAh. The L-tRNAh band was also lost. Thus, RNases present in serum perform a highly efficient conversion of glycine tRNAs into 5′ tRNA halves of precisely 30 or 31 nt, which then accumulate due to their capacity to form RNase-resistant homodimers (22). It remains to be determined whether S-tRNAh are generated by an alternative cleavage site at glycine tRNAs (Figure 4F) or whether they are generated by cleavage or trimming of L-tRNAh. Note that the concentration of tRNA-derived fragments in S+ is below the limit of detection of our assay (Figure 4G). Thus, FBS is solely serving as a source of RNases while not supplying RNAs at a concentration that could interfere with these studies.

### EVs contain full-length ncRNAs while the EV-depleted fraction is enriched in ncRNA fragments.

To evaluate exRNA profiles under standard serum-containing growth conditions (S+; protocol: 1, SI Materials and Methods), we collected CCM at different time points, separated vesicles from nonvesicular RNAs by density gradients and analyzed exRNAs by northern blot.

One striking difference in respect to what was previously observed in ITS incubations (protocol: 3; Figure 4A) or HBSS+ washes (protocol: 4; Figure 3A) was that the tRNA and the 7SL RNA bands were enriched according to what could be expected by rRNA band intensities (Supplementary Figure S3C). Here, addition of RI (120 U; 12 U/ml) did not have any observable effect on exRNA profiles.

Having observed that tRNA^Gly^_GCC_ is particularly susceptible to the action of serum RNases (Figure 4E), we were surprised to detect it by northern blot in the extracellular space of U2-OS cells incubated in the presence of 10% serum (Figure 4G). Furthermore, the intensity of the full-length tRNA band was positively correlated to the length of the incubation. However, density gradient analysis later confirmed that extracellular tRNAs and 7SL RNAs were exclusively associated with EVs (Figure 4H). Conversely, tRNA-derived fragments (glycine S-tRNAh in particular) were only present outside EVs. A similar tendency was found for other RNA polymerase III transcripts like YRNAs and their fragments (Supplementary Figure S3D), in agreement with previous reports in nematodes (37).

In summary, long transcripts (including tRNAs) cannot resist prolonged incubation in samples with high RNase activity such as those containing serum except when protected inside EVs. However, some of their fragments are much more resistant and are consistently detected in the EV-depleted fraction. These fragments define the extracellular nonvesicular RNAome in the absence of added RI, although many of them are not directly released from cells.

### Immunoregulatory potential of extracellular nonvesicular RNAs

We wondered whether dendritic cells (DCs) could sense and react to nonvesicular exRNAs. These cells are regarded as the sentinels of the immune system and link innate and adaptive immune responses (38). Thus, we reasoned that if exRNAs are nonsilent from an immunological perspective, they should be sensed by DCs in the first place.

**Figure 5.**
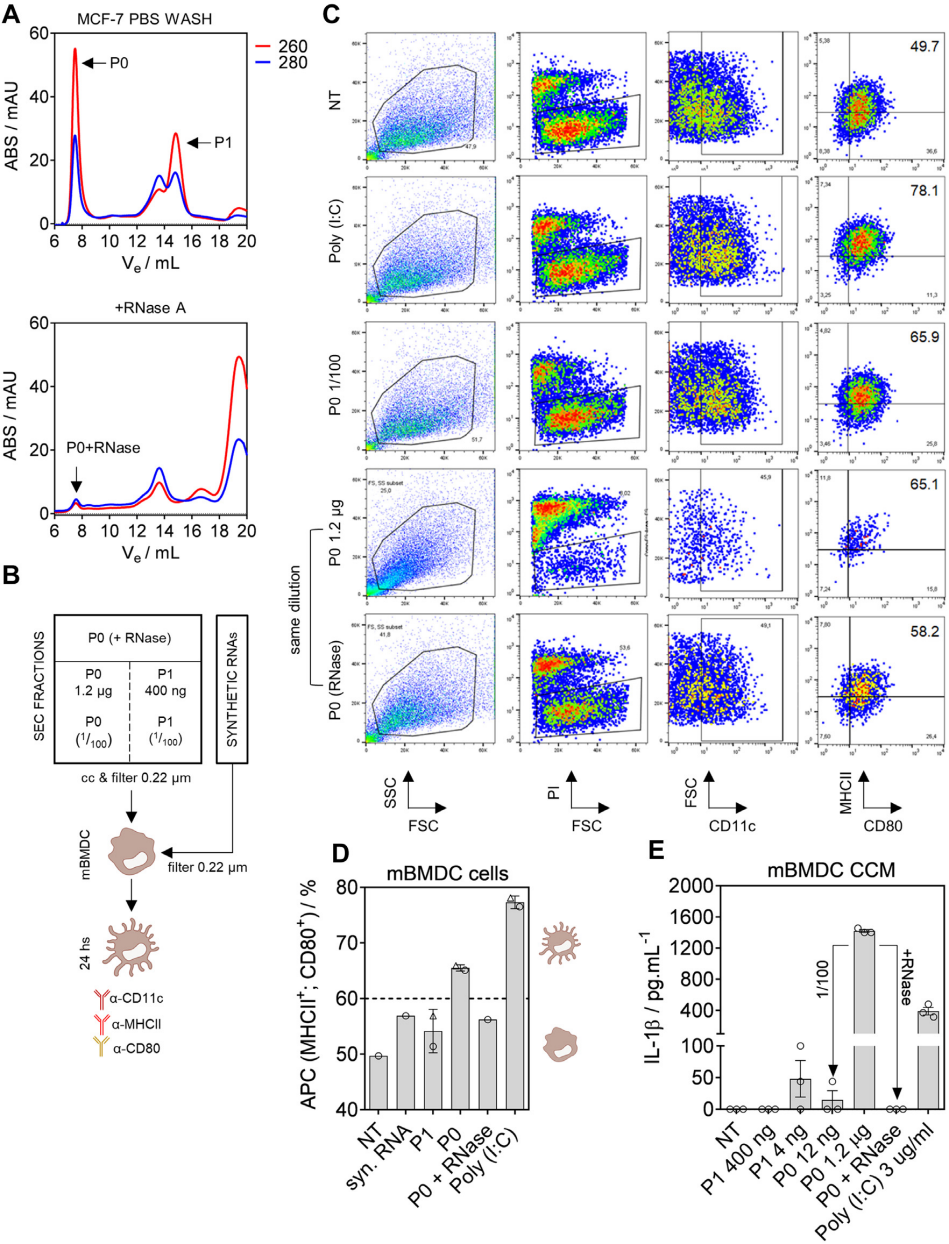
The contents of the P0 peak can trigger dendritic cell maturation in an RNA-dependent manner. (**A**) SEC separation of the P0 and P1 peaks used for dendritic cell maturation assays. Cell-conditioned PBS was concentrated and separated into two aliquots, one of which was treated with RNase A before SEC. (**B**) Concentrated and sterilized SEC fractions were incubated with nonprimed murine bone marrow-derived dendritic cells (BMDC). The TLR3 agonist Poly (I:C) was used as a positive control. (**C**) Flow cytometry analysis of BMDC at *t* = 24 h post exposure to the P0 and P1 peaks (or synthetic RNAs). PI: propidium iodide. FSC: forward scatter. SSC: side scatter. Numbers to the right correspond to the percentage of viable (PI negative), CD11c-positive cells expressing high levels of class II MHC and CD80. (**D**) Percentage of matured BMDC (considered as antigen-presenting cells, APC) at *t* = 24 h post exposure. Triangles correspond to diluted fractions (P0: 1/100; P1: 1/100; Poly [I:C]: 1/10). E) Quantitation by ELISA of IL-1β levels in the media of BMDC analyzed by flow cytometry in the previous panel.

ExRNAs obtained from MCF-7 cells were either treated or not with RNase A and later separated by SEC in order to obtain the following samples/fractions: P0, P0 post RNase-treatment and P1. These fractions were concentrated, filtered and added directly to the media of freshly prepared non-primed bone marrow-derived murine dendritic cells (BMDC) (Figure 5A, B). The synthetic dsRNA analogue Poly (I:C) was used as a positive control. After incubation for 24 h, BMDC maturation was evaluated by flow cytometry monitoring the percentage of CD11c-positive cells expressing high levels of the activation-induced markers MHC class II and CD80 (Figure 5C and Supplementary Figure S4). Interestingly, the purified P0 peak diluted to an RNA concentration as low as 12 ng/ml was sufficient to trigger BMDC maturation, at levels comparable to those obtained when adding micrograms of Poly (I:C) (Figure 5D). Undiluted P0 (1.2 μg/ml) was highly cytotoxic, with more than 90% of cells staining positive for PI. Furthermore, high levels of the pro-inflammatory cytokine IL-1β were found in the media of these cells (Figure 5E). Altogether, these observations suggest that components in the P0 peak can be sensed by DCs when present in the extracellular space. They can trigger DC maturation and, at higher concentrations, a form of cell death presumably related to over activation or pyroptosis. The latter effect is dependent on RNA because undiluted RNase-treated P0 did not induce IL-1β release nor triggered significant BMDC maturation in viable cells. These observations strongly argue against potential endotoxin contamination of the P0 fraction that may have led to DC maturation and/or IL-1β secretion. These results afford new avenues in the biological characterization of exRNAs, suggesting at least some of these RNAs are immunologically nonsilent. This supports the possibility of an immune surveillance mechanism involving exRNAs or RNP complexes such as extracellular ribosomes.

## DISCUSSION

A substantial fraction of exRNAs are not encapsulated inside EVs, yet the extracellular nonvesicular RNAome has not been studied in a comprehensive manner yet. By inhibiting extracellular RNases, our results highlight that the nonvesicular exRNA fraction is highly dynamic. This experimental approach enabled us to obtain exRNA profiles with an unprecedented level of detail and with temporal resolution. Furthermore, we succeeded in stabilizing extracellular full-length tRNAs and ribosomes, which have not been identified before outside EVs due to their susceptibility to extracellular ribonucleases. In contrast, some of their fragments were found to be highly stable and they collectively define the nonvesicular RNAome under standard conditions, especially in the presence of serum. These results have profound implications on the way we understand the mechanisms responsible for RNA release.

The presence of ribosomal aggregates in the extracellular EV-depleted fraction is further supported by the co-isolation of rRNAs, ribosomal proteins and polyA+ mRNAs from the same chromatographic fractions. Extracellular ribosomes were described in the 70’s in the blowfly *Calliphora vicina* (39) but subsequently linked to an experimental artefact (40) and have received little attention since then. However, we have demonstrated that extracellular ribosomes exist at least transiently in the media of cultured mammalian cells and possibly also in body fluids. In support of the latter, a modified small-RNA sequencing method has been recently reported that permits the identification of mRNA fragments in blood plasma or serum (41). Strikingly, the authors found that the distribution and length of reads mapping to mRNAs was reminiscent of ribosome profiling, suggesting that the sequenced fragments could be the footprints of ribosomes circulating in biofluids.

There is an increasing body of evidence showing that EVs actually contain more full-length ncRNAs than microRNAs or ncRNA fragments. For instance, Bioanalyzer's peaks corresponding to intact 18S and 28S rRNAs have been identified in purified EVs (32,42–45), while full-length YRNAs and other ncRNAs have been identified by sequencing, RT-qPCR and/or Northern blot (17,46). The use of thermostable group II intron reverse transcriptases (TGIRT-seq) has allowed the identification of full-length tRNAs in EVs, which greatly outnumber tRNA-derived fragments (47–49). Our results are consistent with these reports, and clearly show the presence of tRNAs and 7SL RNAs in EVs purified by buoyant density flotation in linear iodixanol gradients. At the level of sensitivity achievable by DIG-based Northern blotting, tRNA-derived fragments were not detectable in EVs (Figure 4H). Therefore, TGIRT-seq does not seem to be biasing results toward full-length tRNAs (48), and gives a picture of the RNA content of EVs which is in good agreement with our Northern blot results. This can change, however, when EVs are purified from stressed cells. Considering stressed cells upregulate tRNA-derived fragments (23,24) and that changes in intracellular RNA profiles are mirrored in EVs (50), it is reasonable to speculate that EVs coming from stressed cells could contain higher payloads of stress-induced tRNA halves (51).

**Figure 6.**
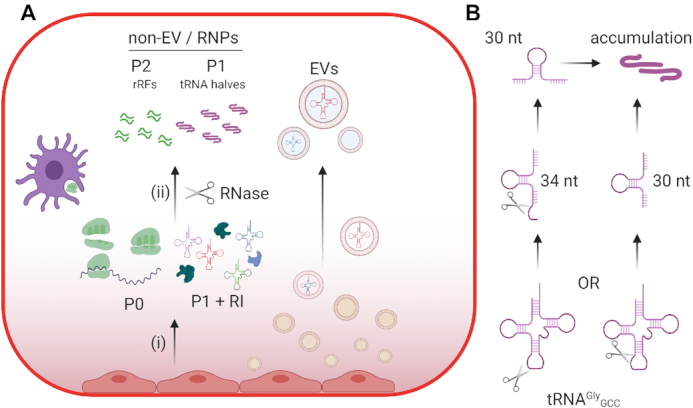
Proposed model. (**A**) Cells in culture release tRNAs, ribosomal subunits or ribosomes to the extracellular space, even outside EVs. When the CCM is analyzed by SEC, these RNAs define the P0 and P1 peaks, respectively (i). However, their detection is only possible after addition of RI to the medium. Active secretion (e.g., autophagy-dependent) might contribute to nonvesicular exRNA profiles, but damaged or dead cells with compromised plasma membrane integrity may be quantitatively more important. Extracellular RNases degrade nonvesicular RNAs and generate stable fragmentation products like glycine tRNA halves (ii), which can assemble into dimers and elute in the chromatographic P1 peak even in the absence of RI. The P2 peak is probably composed of rRNA-derived fragments (rRFs) forming tightly bound dsRNAs which are not amenable to standard small RNA sequencing techniques. While full-length tRNAs and YRNAs are not detected in the non-EV fraction in the absence of RI, those which are present inside EVs are protected from degradation. Thus, EVs are probably the only source of full-length ncRNAs in RNase-rich extracellular samples. Overall, this diagram represent the remarkable differences between what is sequenced in the extracellular space in the absence of RI and what cells actually release, as revealed by RI-SEC-seq. (**B**) A diagram explaining possible biogenetic routes for extracellular, nonvesicular tRNA^Gly^_GCC_ 5’ halves.

Nonvesicular exRNAs have received very little attention (15) until recently (32). In the past, we have compared the small RNA content between EVs and 100,000 x g supernatants of cell-conditioned medium and found that the EV-depleted fraction was highly enriched in 5′ tRNA halves of precisely 30 or 31 nucleotides and almost exclusively derived from glycine or glutamic acid tRNAs (16). Similar results were obtained by other groups working on primary cultures of glioblastoma (17). Furthermore, glycine tRNA halves are predominantly found outside EVs in serum (20) and are ubiquitous in many biofluids including serum, urine, saliva and bile (18). We are now showing that these fragments are not directly released from cells *in vitro*. Instead, they are generated in the extracellular space (Figure 6). Enrichment of these fragments, especially when found in the EV-depleted fraction, is probably a consequence of their differential extracellular stability rather than their preferential or selective secretion. This is further supported by the recent observation that circular RNAs, which are known to be highly stable, are increased in nearly all human biofluids when compared to matched tissues (52).

Live cells can release a representative fraction of their cytoplasm by mechanisms such as cytoplasmic extrusion (53) or amphisome fusion with the plasma membrane (32), and these mechanisms could be involved in the release of nonvesicular ribosomes and tRNAs to the extracellular space. However, a few events of cellular damage or death might be quantitatively more important in defining exRNA profiles as has been discussed above. In support of this, it has been shown that extracellular rRNA levels correlate with extracellular lactate dehydrogenase (LDH) activity, which is widely used as a marker of cell death (54). Even though exRNA analysis derived from dead cells can be considered as an artifact of cell culture, there are situations where nonapoptotic, immunogenic cell death (ICD) occurs at abnormal frequencies in an organism. These situations include aging (55), trauma (56), ischemia-reperfusion injury (57), infectious diseases and cancer. In the latter, ICD can occur because of the hypoxic inner mass characteristic of solid tumors or following treatment with cytotoxic agents (58). In all cases, dying cells release intracellular components which are sensed by innate immune cells and interpreted as damage-associated molecular patterns (DAMPs). Furthermore, the therapeutic activity of several anticancer drugs eliciting ICD involves an autocrine and paracrine circuit which depends at least in part on the release of self RNAs by stressed or dying cancer cells (59). Because rRNAs and tRNAs are highly abundant intracellularly and they are exposed in the extracellular space in cases of damage, and considering RNAs are actively sensed by the innate immune system (60,61), we hypothesized that exRNA-containing nonvesicular complexes could be endowed with immunomodulatory abilities. The high turnover of these complexes as a consequence of extracellular RNases could prevent activation under physiological conditions.

As for the ribonuclease responsible for extracellular, nonvesicular tRNA cleavage, it is clearly a serum-derived ribonuclease in FBS-containing samples (probably RNase A itself). When serum is not present or highly diluted, such as after thoroughly washing cells with serum-free media or buffers, it is possible that endogenous secreted RNases are responsible for shaping nonvesicular exRNA profiles. Stressed cancer cells secrete enzymes to perform some metabolic reactions in the extracellular space and then uptake the enzymatic products to fuel cellular energetics (62). By analogy, we are tempted to speculate that secreted RNases such as ANG could play a role in extracellular RNA metabolism, preventing the toxicity associated with their intracellular activity in nonstressed cells (63). Although the function of ANG in tRNA cleavage seems to be partially redundant (36,64), its implications in extracellular RNA cleavage under physiological conditions remains to be elucidated. Redundancy might be lower in serum-free environments as the nervous system, where several mutations in ANG have been functionally linked to neurodegenerative diseases (65). We have provided preliminary evidence suggesting an involvement of extracellular, nonvesicular RNAs or RNPs in immune surveillance. Thus, a link between mutations in ANG and deregulated extracellular RNA fragmentation patterns is feasible in diseases such as ALS whose etiology or evolution is deeply connected to inflammation (66).

Bacterial rRNA and tRNAs induce Toll-like receptor (TLR)-dependent DC maturation and IL-1β secretion, and are therefore considered pathogen-associated molecular patterns. However, to elicit such a response, addition of the purified RNAs with cationic lipids seems to be essential (67). In contrast, we have obtained high extracellular levels of IL-1β when incubating BMDC with approximately one microgram of RNA obtained from the P0 peak of MCF-7 cells (composed mainly of ribosome particles) in the absence of any transfection reagent. Strikingly, this effect was lost when incubating DCs with RNase A-pretreated P0. It remains to be elucidated whether RNA itself or any potentially associated RNA-binding proteins are responsible for these effects. We address some limitations in our experimental design, including the incubation of human-derived RNAs with murine dendritic cells. Thus, these results should be interpreted with caution. Follow-up studies will confirm whether or not extracellular nonvesicular RNAs are active players in immune surveillance.

Although extracellular ribosomes are not predicted to resist extracellular RNases they might still achieve functionally relevant concentrations *in vivo* in extracellular microenvironments. The identification of a RNase-resistant peak (P2) derived from partial fragmentation of P0 (and possibly P1 as well) suggests that, similarly to what we have shown for 30–31 nt tRNA halves, rRNA-derived fragments may accumulate in the extracellular space and their extracellular concentration may increase in situations of abnormal cell death. A new method has been recently described enabling RNA sequencing from a few microliters of human serum (68). With this method, almost perfect separation between normal and breast cancer patients was possible based on rRNA or mitochondrial tRNA sequences.

In conclusion, ribonuclease inhibition dramatically shapes extracellular RNA profiles and uncovers a population of extracellular ribosomes, tRNAs and other coding and noncoding RNAs. These RNAs, which are not protected by encapsulation inside EVs, are rapidly degraded by extracellular RNases. However, some of their fragments resist degradation and can accumulate in cell culture media and in biofluids. This dynamic view of exRNAs impacts our understanding of RNA secretion mechanisms and may offer a window to new molecules with biomarker potential. These include intrinsically stable ncRNA fragments and extracellular RNPs stabilized by addition of RI immediately upon collection of samples.

## NOTE ADDED IN REVISION

During the review process of this manuscript, an article has been accepted for publication in Nucleic Acids Research which has shown that human RNase 1 generates tRNA- and YRNA-derived fragments in the extracellular space and outside EVs (69). This report supports many of our findings. Taken together, the support for the extracellular biogenesis of extracellular small RNAs seems to be strong.

## DATA AVAILABILITY

Data is available at NCBI SRA under the BioProject ID: PRJNA633249.

## Supplementary Material

gkaa674_Supplemental_Files
