# Supplementary material for: Fragmentation of extracellular ribosomes and tRNAs shapes the extracellular RNAome
Source: Nucleic Acids Res. 2020 Aug 12;48(22):12874–88. doi: 10.1093/nar/gkaa674 (PMC7736827; doi:10.1093/nar/gkaa674)
Supplement: gkaa674_Supplemental_Files [file gkaa674_supplemental_files.zip › Supplementary Methods_R2.docx]

**Fragmentation of extracellular ribosomes and tRNAs shapes the extracellular RNAome**

Juan Pablo Tosar^1,2,3^*; Mercedes Segovia^4^; Mauricio Castellano^1,2^; Fabiana Gámbaro^2,5^; Yasutoshi Akiyama^3^, Pablo Fagúndez^1,2^, Álvaro Olivera^6^, Bruno Costa^1,2^, Tania Possi^2^, Marcelo Hill^4^, Pavel Ivanov^3,7^, Alfonso Cayota^2,8^*.

^1^Analytical Biochemistry Unit. Nuclear Research Center. Faculty of Science. Universidad de la República, Uruguay. ^2^Functional Genomics Unit, Institut Pasteur de Montevideo, Uruguay. ^3^Division of Rheumatology, Immunology and Allergy, Brigham and Women's Hospital, Boston, MA, USA. Department of Medicine, Harvard Medical School, Boston, MA, USA. ^4^Laboratory of Immunoregulation and Inflammation, Institut Pasteur de Montevideo, Uruguay. Immunobiology Department, Faculty of Medicine, Universidad de la República, Uruguay. ^5^Molecular Virology Laboratory, Nuclear Research Center. Faculty of Science. Universidad de la República, Uruguay. ^6^Centro Universitario Regional Este, Universidad de la República, Uruguay. ^7^The Broad Institute of Harvard and M.I.T., Cambridge, MA, USA. ^8^Department of Medicine, University Hospital, Universidad de la República, Uruguay.

*To whom correspondence should be addressed. Email: jptosar@pasteur.edu.uy; cayota@pasteur.edu.uy

**SUPPLEMENTARY INFORMATION: MATERIALS AND METHODS**

**LEGENDS TO SUPPLEMENTARY FIGURES**

**Supplementary Figure 1:** data associated to Figure 1. A) Injection of synthetic RNAs of 30 nt corresponding to 5’ tiRNA^Gly^_GCC_(which forms RNA dimers as reported in Tosar et al. (2018) ^22^; red) and a mutant with a 25U/C substitution (which is not able to dimerize; violet) in a Superdex 200 10/300 column with PBS 1x as the mobile phase. B-C) same as Figure 1(H) and Figure 1(I) but in the P1 peak of MCF-7 CCM either treated (top) or not treated (bottom) with RI. D) Representation of the predicted SNORD49A (U49A; black)/28S rRNA (red) interaction, as depicted in snoRNABase (www-snorna.biotoul.fr). Below is the sequence with the highest number of reads. Its relative abundance is expressed as reads per million mapped reads (RPM). Its ranking in the “P1 + RI” dataset is also shown. E) Alignment of reads mapping to tRNA^Glu^ (anticodons CUC and UUC) and the genomic sequence for tRNA^Glu^_UUC_with manual addition of the 3’ CCA sequence. “A.C”: anticodon. F) Coverage plots of sequences mapping to 28S rRNA in P0 (red), “P1 + RI” (green), “P1 – RI” (violet) and P2 (blue), either in linear scale (top) or Log2 scale (bottom). G) Coverage plot of sequences mapping 5.8 S rRNA.

**Supplementary Figure 2:**data associated to Figure 2. A) Same as Figure 2(B) but washing cells with MEGM instead of PBS. B) Deconvolution of chromatograms obtained by SEC analysis of PBS washes of different adherent malignant and nonmalignant cell lines, derived from different mammalian species. C) U2-OS cells before (left) and after (right) four consecutive washes with HBSS for 30 seconds.

**Supplementary Figure 3:**data associated to Figure 4. A) Experimental conditions were similar to those used in Figure 4 A-D, but the cell-free processing step was omitted. Control lanes include RNA lysates from U2-OS cells incubated in DMEM + 10% FBS (S+) or in the same cells used for exRNA analysis (ITS, 1 hour). L: 5’ tRNA halves of 33 – 34 nt; S: 5’ tRNA halves of 30 – 31 nt. B) Comparison of SYBR gold-stained denaturing 10% PAGE gels from Figure 4, C and Supplementary Fig. 4, A. A parameter named the RNA Degradation Number (RDN) was defined as the ratio between SYBR gold intensities above and below the tRNA band. The lower the RDN (the higher extent of extracellular fragmentation), the higher the fragment-to-full-length-tRNA ratio (estimated by densitometric analysis of Northern blot bands). C) Effect of RI addition (120 U in 10 mL) in exRNA profiles from U2-OS CCM (1 hour in ITS medium or 1 – 24 hours in S+ medium). D) Analysis of YRNAs (left) or some selected 5’ fragments (right) by RT-qPCR (left) or SL-RT-qPCR (right) in 100,000 x g pellets (EVs) or concentrated 100,000 x g supernatants (RNPs) of U2-OS ΔANG conditioned medium (t = 48 hs; MEGM).

**Supplementary Figure 4:** data associated to Figure 5C. Complete flow cytometry analysis in all the samples included in Figure 5, D-E. Gly (31): a synthetic single-stranded RNA of 31 nucleotide with the sequence of glycine 5’ tRNA halves.

**SUPPLEMENTARY METHODS**

**Reagents**

RI (ribonuclease inhibitor, murine; 40U / µL) was purchased from New England Biolabs. Sterile Phosphate buffered saline, PBS (10x DPBS for chromatographic separations, 1x DPBS with or without calcium and magnesium for cell culture), DMEM, trypsin–EDTA solution, fetal bovine serum (FBS), 100x Insulin-Transferrin-Selenium solution (ITS) and nuclease-free distilled water were obtained from Gibco. Hank’s Balanced Salt Solution (HBSS, no phenol red), either with or without calcium and magnesium were obtained from Corning as 1x sterile solutions. Trizol and Trizol LS reagents were from Invitrogen (Thermo). The 60% Optiprep solution used to prepare iodixanol gradients was obtained from Sigma.

**Cell lines and preparation of cell-conditioned medium/buffers.**

Cell lines were obtained from ATCC. Creation and characterization of U2-OS ΔANG and U2-OS ΔRNH1 cells was described in Akiyama et al. 2019 (doi: 10.1101/811174). The gene-edited cells were clonally selected and genotyped.

Conditioned medium was typically derived from one 75 cm^2^ flask for chromatographic analysis or from one 150 cm^2^ flask for electrophoresis. Cells were plated at a density which was adjusted to obtain a confluency of 80% at the endpoint of the experiment.

Extracellular samples containing RNAs were prepared based on the following protocols:

Preparation of serum-containing conditioned medium (protocol 1): cells were grown in DMEM + 10% FBS (“S+” medium). They were washed with S+ medium, and incubated in S+ for variable periods of time which ranged from 1 to 48 hours.

Preparation of serum-free conditioned medium (protocol 2): cells were plated on day zero and grown in S+ for 24 hours. Later, they were washed with PBS, grown in Mammary Epithelial Growth Medium without antibiotics and without bovine pitutary extract (MEGM, Lonza) for 48 hours, washed again with PBS, and grown in MEGM for additional 48 hours.

Preparation of serum-free conditioned medium (protocol 3): cells were grown in S+, washed with DMEM and incubated with DMEM + 1x Insulin-Transferrin-Selenium solution (“ITS” medium) for one hour.

ExRNA analysis after short washes in PBS or Hank’s Balanced Salt Solution (HBSS) (protocol 4): cells were grown in S+ medium until 80% confluent, washed three times with warm buffer (5 seconds per wash), and washed a forth time for 30 seconds with 5 mL (in T75 flasks) or 10 mL (in T150 flasks) of warm buffer plus 20 – 40 U RI (respectively). Buffers could correspond to PBS, PBS plus divalent cations (PBS+), HBSS or HBSS plus divalent cations (HBSS+) depending on the experiment. Flasks were gently tilted from side to side during washes.

For protocols 1-4: conditioned media or conditioned-buffers were centrifuged at 800 x g at room temperature to remove detached cells and then spinned twice at 4°C and 2,000 x g. The supernatants were either processed immediately or stored at -20°C for later use. If frozen, media was spinned again at 4°C and 2,000 x g upon thaw.

**Antibodies**

The following primary antibodies were used for Western blot: anti-Puromycin (Millipore, cat # MABE343, clone 12D10); anti-RPS6 (Santa Cruz Biotechnology; cat # sc-74459; clone C-8); anti-RPS23 (Santa Cruz Biotechnology; cat # sc-100837; clone SJ-K2); anti-RPL7a (Cell Signaling; cat # 2415; clone E109); anti-CD63 (BD Biosciences; cat # 556019; clone H5C6); anti-CD9 (Millipore; cat # CBL162; clone MM2/57); anti-CD81 (R&D; cat # MAB4615; clone 454720); anti-eIF2α (Cell Signaling: cat # 9722); anti-(p)eIF2α (Ser51; Cell Signaling; cat # 9721). Antibodies for flow cytometry where from BD and directed to mouse proteins CD11c (clone HL3), CD80 (clone 16-10A1), I-Ab (clone AF6-120.1).

**Primers and RT-qPCR**

In figure 1J: cDNA was obtained with SuperScript II (Thermo) using random hexamers and following manufacturer’s instructions. For glycine 5’ halves a gene-specific primer (GSP) was used (the sample was heated at 65^o^C, following by primer annealing and extension at 42^o^C). Input material: 2 µL of each chromatographic fraction. Quantitative real-time PCR was performed with a Kapa SYBR Fast qPCR Master Mix (2x) from Kapa Biosystems.

tRNA^Gly^ 5’ halves (RT GSP): TGCCATCCACCACCCTGTTGCTGTAGCGAGAATT

tRNA^Gly^ 5’ halves (F-primer): ccCCGCATTGGTGGTTCAGTGGTA

tRNA^Gly^ 5’ halves (R-primer): TCCACCACCCTGTTGCTGTA

28S rRNA (position 310; F-primer): GGGTGGTAAACTCCATCTAAGG

28S rRNA (position 310; R-primer): GCCCTCTTGAACTCTCTCTTC

28S rRNA (position 3744; F-primer): GTAAACGGCGGGAGTAACTATG

28S rRNA (position 3744; R-primer): GACAGTGGGAATCTCGTTCATC

18S rRNA (position 442; F-primer): CTGAGAAACGGCTACCACATC

18S rRNA (position 442; R-primer): GCCTCGAAAGAGTCCTGTATTG

5.8S rRNA (position 25; F-primer): CTCGTGCGTCGATGAAGAA

5.8S rRNA (position 25; R-primer): TCGAAGTGTCGATGATCAATGT

5.8S rRNA (position 2; F-primer): ACTCTTAGCGGTGGATCACT

5.8S rRNA (position 2; R-primer): GATGATCAATGTGTCCTGCAATTC

In Figure 3E: Chromatographic fractions corresponding to the P0 peak from BJ cells were concentrated to 50 µL by ultrafiltration (Vivaspin 500; MWCO 5 kDa). One microliter was used as input. cDNA was obtained with SuperScript II (Thermo) using an oligo(dT)_18_ primer and following manufacturer’s instructions. End-point PCR was performed with recombinant Taq DNA Polymerase (Thermo) for 35 cycles. Annealing temperature: 58^o^C. Expected amplicon length: 731 bp ( > 2200 bp from predicted mRNA 3’ end)

HSP90B1 (F-primer): GGTGTAGGAATGACCAGAGAAG

HSP90B1 (R-primer): GGAGCAGATGTGGGTACAAATA

In Supplementary Figure S4, F: cDNA for full-length YRNA analysis was obtained with SuperScript II (Thermo) using random hexamers and following manufacturer’s instructions. cDNA for YRNA fragments was obtained based on the stem-loop RT-qPCR method which was described in detail in our previous publication (Tosar et al. 2018; *Nucleic Acids Research* 46, 9081-9093).

Full-length YRNAs (conventional RT with random hexamers):

RNA Y1 (F-primer): TGGTCCGAAGGTAGTGAGTTA

RNA Y1 (R-primer): GTCAAGTGCAGTAGTGAGAAGG

RNA Y3 (F-primer): CCGAGTGCAGTGGTGTTTA

RNA Y3 (R-primer): AGGGCTAGTCAAGTGAAGCAG

RNA Y4 (F-primer): GTCCGATGGTAGTGGGTTATC

RNA Y4 (R-primer): AAAGCCAGTCAAATTTAGCAGT

RNA Y5 (F-primer): GTCCGAGTGTTGTGGGTTATT

RNA Y5 (R-primer): ACAGCAAGCTAGTCAAGCG

YRNA fragments (SL-RT-qPCR; as described in Tosar et al. 2018):

Stem-loop RT primer (“X” denotes assay-specific 3’ overhangs): GTCGTATCCAGTGCAGGGTCCGAGGTATTCGCACTGGATACGACXXXXXX

RNA Y1 (5’ fragment; 31 nt; 3’ overhang): ATTGAG

RNA Y4 (5’ fragment; 32 nt; 3’ overhang): AGTTCT

RNA Y5 (5’ fragment; 32 nt; 3’ overhang): CTTAAC

miR-21-5p (3’ overhang): GTCAAC

RNA Y1 (5’ fragment; 31 nt; F-primer): caagTGGTCCGAAGGTAGTGAGT

RNA Y4 (5’ fragment; 32 nt; F-primer): tgGTCCGATGGTAGTGGGTT

RNA Y5 (5’ fragment; 32 nt; F-primer): agttgGTCCGAGTGTTGTGG

miR-21-5p (F-primer): gccccgTAGCTTATCAGACTGATGT

Universal reverse primer: GTGCAGGGTCCGAGGT

All primers were obtained from Integrated DNA Technologies (IDT, USA).

**Density gradient separations**

For separation of ribosomal subunits and ribosomes, a linear gradient composed of 10 – 40 % RNase-free sucrose was used. The gradients were prepared in 20 mM Tris-Cl buffer (pH = 8), 4 mM MgCl_2_, 50 mM KCl and 1mM DTT (added fresh). Layers of 40%, 30% 20% and 10% sucrose were added sequentially to a 12 mL polypropylene tube and frozen at -80^o^C in between. The whole gradient was thawed overnight in the cold-room and used the next day. Extracellular samples were obtained from U2-OS cells using protocol 4 and washing with HBSS+ in the presence of RI. Washes four and five were pooled, concentrated by ultrafiltration and stored at -80°C until use. Concentrated extracellular fractions (0.5 mL) were thawed, layered gently on top of the gradient, and centrifuged at 186,000 x g for 3 hours at 4^o^C using a SW 40 Ti rotor (acceleration: max; break: min). A density gradient analyzer and fractionator equipped with a Teledyne ISCO UA-6 UV/Vis detector was used to collect fractions of 0.5 mL, starting from the top of the gradient. Collected fractions were treated with 0.5 mL TRIzol to purify both RNA and proteins according to the manufacturer’s instructions.

To separate extracellular vesicles from ribonucleoproteins (RNPs) or other high-density components of the non-EV fraction, high-resolution (12 – 36 %) iodixanol gradients were used following the protocol described in ^23^. Briefly, samples were layered on the bottom of a 12 mL polypropylene tube, and layers of ice-cold 36%, 30%, 24%, 18% and 12% iodixanol (prepared in PBS) were added sequentially on top. The gradients were centrifuged for 15 h at 120,000 x g and 4^o^C, using a SW40 Ti rotor. Twelve fractions of 1 mL were obtained from the top of the gradient and transferred to new tubes. One half of each fraction was treated with an equal volume of TRIzol and used for RNA and protein purification following manufacturer’s instructions. The other half was twice precipitated with cold (-20^o^C) 60% acetone, and the pellets were resuspended in 1x loading buffer for Western blot analysis. Loading buffer contained reducing agents or not depending on the primary antibodies intended to use.

**Sequencing data analysis**

After sample demultiplexing and adapter trimming (only sequences > 15 bases which contained an identifiable 3′ adaptor were analyzed), FastQ files containing sequencing information were mapped to the human genome (hg38) with RNA STAR (--outFilterMultimapNmax=2000, to allow mapping to repetitive tRNA and rRNA genes, --outFilterMismatchNoverLmax=0.25, --alignIntronMax=30, and default settings). To determine read length distribution counts for RNA biotypes across experimental samples, mapped reads were filtered with BAM-filter (-mapped=TRUE, -include bed and default settings) using small nuclear RNA, small nucleolar RNA, vault RNA, YRNA (Ensemble release 100), mature miRNA (miRbase release V22), tRNAs (UCSC) and ribosomal RNA (custom GTF) genomic coordinates. Filter reads were extracted, converted to Fasta and the length of each individual read was computed. The relative abundance of reads with a given length was expressed as reads per million (RPM) mapped reads by dividing its absolute count number by the total amount of mapped reads in the data set and multiplying by a million. rRNA 28s coverage was determined with bamCoverage (--minMappingQuality=0, --region=chr21:8213887:8218941 and default settings). The relative abundance of reads mapping to different tRNA isoacceptors was determined with feature counts (-M, -- fraction=TRUE, default settings). Data was submitted to NCBI’s small read archive (SRA) under the BioProject ID: PRJNA633249.

**Northern blotting**

RNA samples were run on 10% TBE-urea polyacrylamide gels (ThermoFisher Scientific), transferred to positively charged nylon membranes (Roche). The membranes were cross-linked by UV irradiation. After cross-linking, the membranes were hybridized overnight at 40°C with digoxigenin (DIG)-labeled DNA probes in DIG Easy Hyb solution (Roche). After low stringency washes (washing twice with 2× SSC/0.1% SDS at room temperature) and a high stringency wash (1× SSC/0.1% SDS at 40ºC), the membranes were blocked in blocking reagent (Roche) for 30 min at room temperature, probed with alkaline phosphatase-labeled anti-digoxigenin antibody (Roche) for 30 min, and washed with 1x TBS-T. Signals were visualized with CDP-Star ready-to-use (Roche) and detected using ChemiDoc imaging system (BioRad) according to the manufacturer’s instructions. Oligonucleotide probes were synthesized by IDT. DIG-labeled probes were prepared using the DIG Oligonucleotide tailing kit (2nd generation; Roche) according to the manufacturer’s instructions. The sequences of the probes were as follows:

probe for 5'-tRNA^Lys^_UUU_ : 5' CTGATGCTCTACCGACTGAGCTATCCGGGC 3';

probe for 5'-tRNA^iMet^_CAU_ : 5' CTTCCGCTGCGCCACTCTGCT 3';

probe for 5'-tRNA^Gly^_GCC_ : 5' CTACCACTGAACCACCCATGC 3';

probe for 3'-tRNA^Gly^_GCC_ : 5' GCCGGGAATCGAACCCGGGCCTCCCGCG 3';

probe for 7SL RNA: 5' CACTACAGCCCAGAACTCCTGGACT 3'.

**Protein puromycilation assays**

Extracellular samples were obtained from four T150 flasks of U2-OS cells (70% confluency) based on protocol 4 (PBS+ in Figure 3, G and HBSS+ in Figure 3, H) and concentrated by ultra-filtration (cut-off: 10 kDa) to 300 µL, which were then separated in three identical aliquots. Each aliquot received 40 U RI and 80 mM KCl. Puromycin was added to two aliquots at a final concentration of 5 µg / mL. Two aliquots also received ATP and GTP at 1mM each. All samples were incubated at 37^o^C for two hours. Proteins were then precipitated with cold acetone, washed with cold acetone at 60%, and analyzed by Western blot using an anti-puromycin antibody (1 / 1000, overnight, in PBS, BSA 5%, Tween 0.1 %). In a separate experiments, samples were incubated at either 37^o^C or 4^o^C.

**Transmission electron microscopy**

Two T75 flasks containing cells grown in S+ at 80% confluency were washed twice with serum-free DMEM, once with PBS, and once with PBS + 40U RI. This last wash was concentrated and subjected to size exclusion chromatography as previosuly described. The fractions corresponding to the P0 peak were concentrated to 20 µL by ultrafiltration and frozen. Once thawed, samples were incubated on carbon-coated grids for 1.5 min. The grids were washed by touching a miliQ water drop and blotted with filter paper (twice), then two series of touching a 1% phosphotungstic acid drop and blotting with filter paper before air drying. Grids were imaged in a JEOL JEM-2100 electron microscope at 200 kV.

Extracellular samples from U2-OS cells were obtained following protocol #4, concentrated to 20 µL by ultrafiltration and frozen. Once thawed, samples were incubated on carbon-coated grids for 10 min. The grids were washed once by touching and blotting a miliQ water drop, placed inverted on top of a droplet containing 1% uranyl acetate for 1 minute, then grids were washed by touching and blotting a miliQ water drop, air dried and imaged in a JEOL JEM-2100 electron microscope at 200 kV.

**Sample preparation for dendritic cell maturation assays**

Bone marrow cells were obtained from the leg bones of C57BL/6 mice and differentiated in culture for eight days in the presence of 0.4 ng / mL GM-CSF as described in ^26^.

Two T75 flasks containing MCF-7 cells were grown in MEGM for 48 hours (80% confluency) and then washed with PBS + 40U RI for 30 seconds. The cell-conditioned PBS was separated into two aliquotes. One aliquot was treated with 320 ng RNase A and incubated at 37^o^C for 30 min. Both aliquotes were then subjected to SEC in order to obtain the following fractions/samples: P0, RNase-treated P0 and P1. Each of these fractions were concentrated to 100 µL by ultrafiltration (MWCO 5 kDa) and filter-sterilized in the cell culture room. P0 and P1 were also diluted 100-fold with sterile PBS. All samples (100 µL) were added directly to the media of 1 x 10^6^ BMDCs grown in 900 µL of complete media (RPMI + 10% FBS, 0.05 mM 2-mercaptoethanol, 2mM L-glutamine, 1mM sodium pyruvate, 1% HEPES, 100 U/mL penicillin, 0.1 mg/mL streptomycin ; 6-well plates) and incubated for 24 hours at 37^o^C and 5% CO_2_. Control wells contained: 100 µL PBS (NT; nontreated cells), synthetic tRNA^Gly^_GCC_ 5’ halves or a mutated version of this RNA (25^U/C^; both at 10 µg / mL final concentration), or the synthetic dsRNA analogue Poly(I:C) (Invivogen) at either 30 µg / mL or 3 µg / mL, diluted in PBS.

At that time, cells were harvested and resuspended in PSA (PBS, 0.2% fetal bovine serum, 0.1 sodium azide) to stain them with antibodies against CD11c, CD80 and I-Ab for 20 minutes on light-protected ice. After two washes with PBS, propidium iodide was added. The samples were acquired in the CyAN ADP Analyzer (Dako) and the data analyzed with the FlowJo vX.0.7 software (FlowJo, LLC)

Levels of IL-1β in the media were measured using a commercial ELISA kit from Biolegend.
