# Supplementary figures and images for: Fragmentation of extracellular ribosomes and tRNAs shapes the extracellular RNAome
Source: Nucleic Acids Res. 2020 Aug 12;48(22):12874–88. doi: 10.1093/nar/gkaa674 (PMC7736827; doi:10.1093/nar/gkaa674)

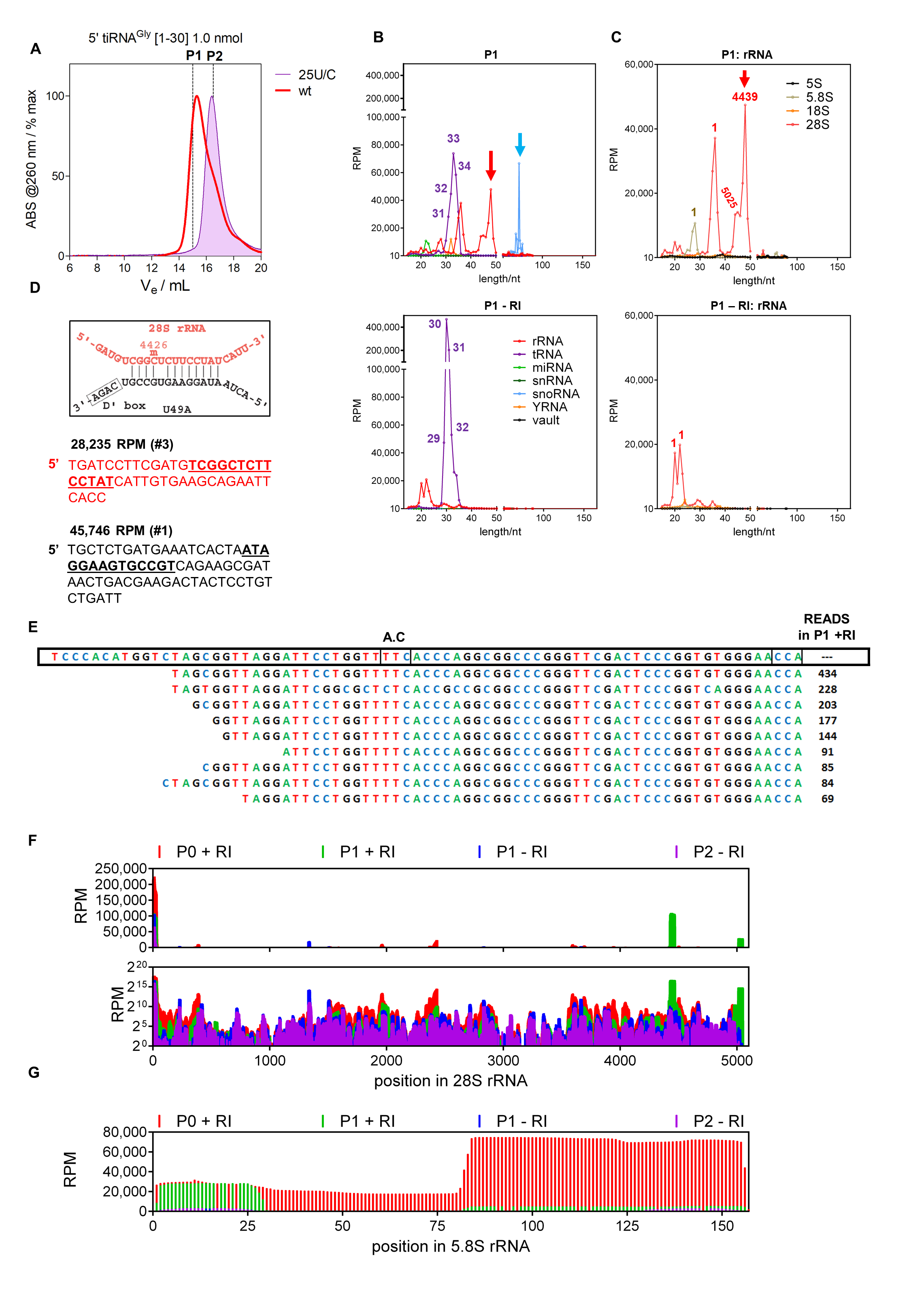

Supplement: gkaa674_Supplemental_Files [file gkaa674_supplemental_files.zip › Figure Sup1_R2.tif]

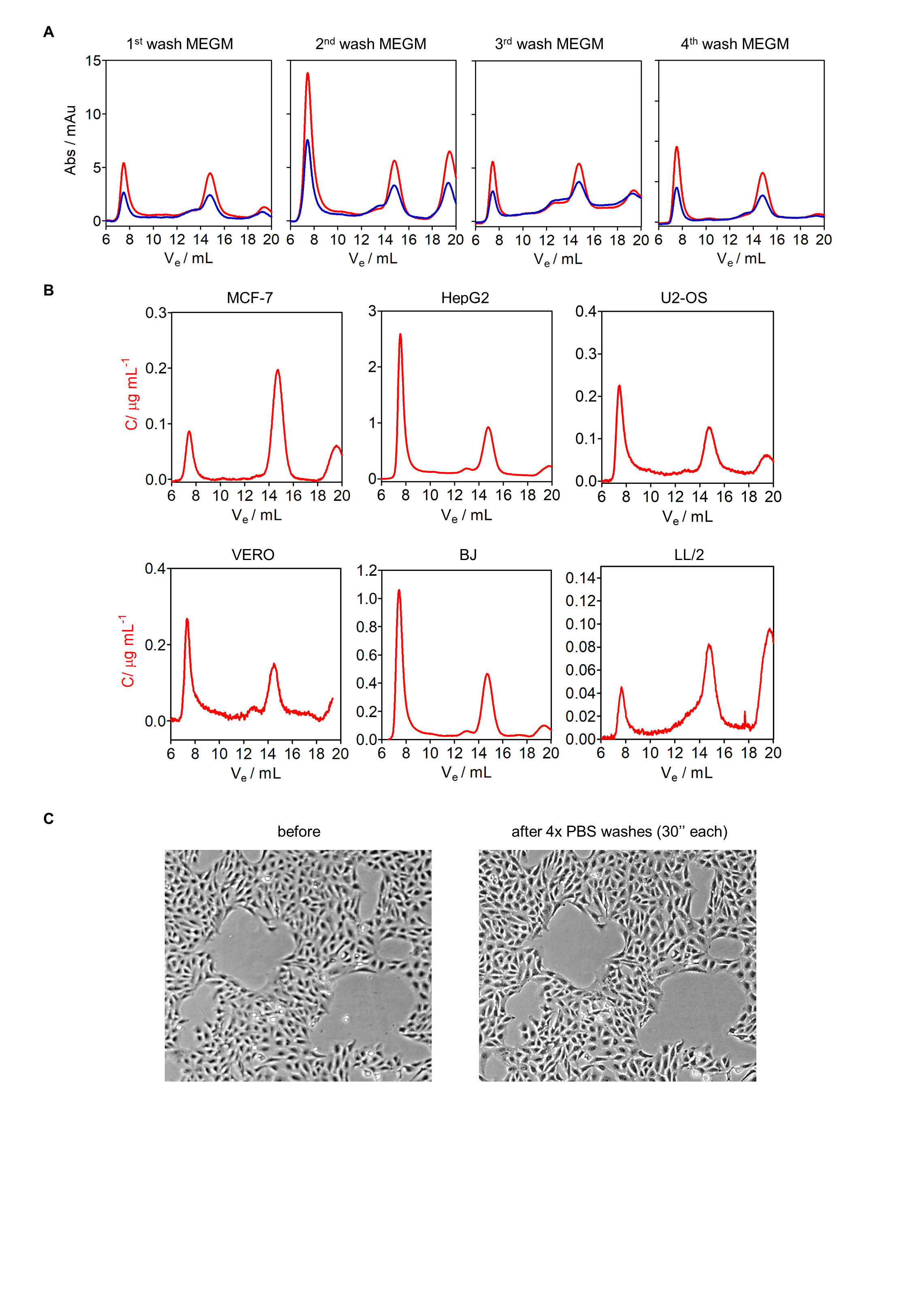

Supplement: gkaa674_Supplemental_Files [file gkaa674_supplemental_files.zip › Figure Sup2_R2.tif]

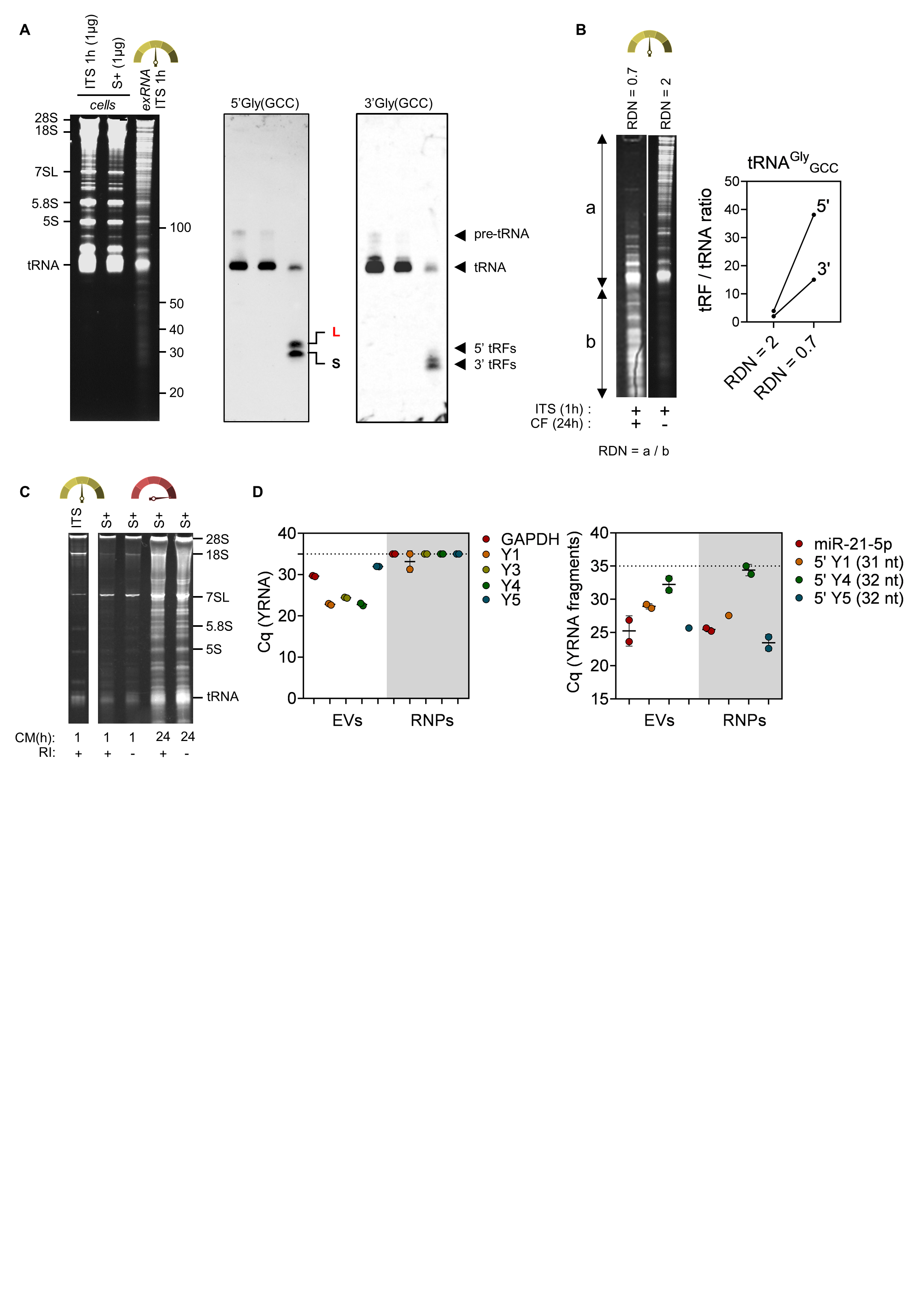

Supplement: gkaa674_Supplemental_Files [file gkaa674_supplemental_files.zip › Figure Sup3_R2.tif]

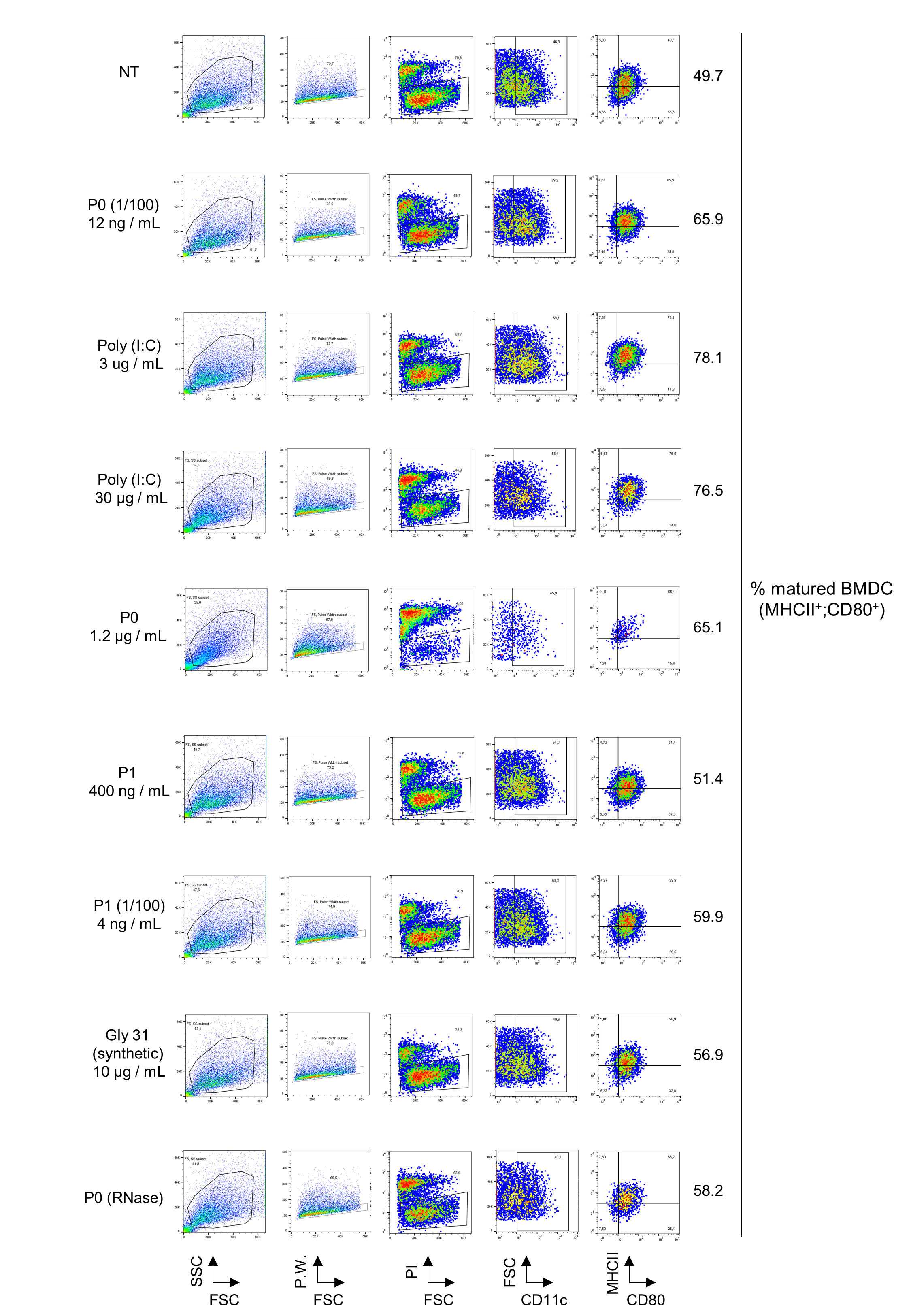

Supplement: gkaa674_Supplemental_Files [file gkaa674_supplemental_files.zip › Figure Sup4_R2.tif]
